# Optimal strategies for contract farming supply chain with government subsidies under e-commerce assistance to farmers

**DOI:** 10.1371/journal.pone.0311490

**Published:** 2024-10-29

**Authors:** Zhengxiang Wu, Jinlei Zhu

**Affiliations:** School of Business Administration, Liaoning Technical University, Huludao City, China; IBS Hyderabad: ICFAI Business School, INDIA

## Abstract

The agricultural issue is a focal point of concern for each country, and e-commerce assistance to farmers, as an emerging model, is gaining increasing attention. Considering this trend, this paper bases on e-commerce assistance to farmers and considers four types of government subsidies: no subsidy, price subsidy model with the farmer as the subsidy target, price subsidy model with the e-commerce platform as the subsidy target, and area subsidy model. Based on this, a game model of the contract farming supply chain involving a farmer and an e-commerce platform was established to explore the optimal decision-making in the contract farming supply chain considering various government subsidies under the background of e-commerce assistance to farmers. The results indicate that: (1) Consumer preference for supporting farmers and consumer premiums can stimulate the farmer to expand farm size, increase agricultural production, increase the purchase price of agricultural products and promote the growth of profits for the farmer and the e-commerce platform, contributing to the growth of social welfare. Output volatility can lead to a decrease in farm size and a reduction in the purchase price of agricultural products. (2) Government subsidies can support the farmer in expanding farm size and contribute to increased profits for the farmer and the e-commerce platform. However, government subsidies do not universally have a positive impact on every variable. The influence of government subsidies on the purchase price of agricultural products is contingent upon the method of subsidy distribution. Similarly, the effects of government subsidies on social welfare are influenced by factors such as price elasticity and agricultural productions per unit area. (3) From the perspectives of farm size, the purchase price of agricultural products, and profits of the farmer and the e-commerce platform, the government will have different and more targeted subsidy models, and the corresponding subsidy models are influenced by the agricultural productions per unit area. The research conclusion can provide references for optimal decision-making in contract farming under the model of e-commerce assistance to farmers.

## 1. Introduction

Agricultural development is the lifeblood of the nation [1, 2]. Taking into account the weaknesses of agriculture itself and the limited access of farmers to information, agriculture faces inevitable hurdles in both production and marketing. In the realm of agricultural production, farmers contend with the adverse impacts of natural disasters, then in agricultural sales, they must navigate the recurrent fluctuations in spot prices [3, 4]. Therefore, they face uncertainties in yield and contend with market failures [5, 6]. This makes it difficult for farmers to cope with unforeseen risks on their own. As a result, the contract farming model has emerged to help farmers cope with the risk of agricultural yield uncertainty and market risk. Contract farming is an agreement between the grower and the processor or retailer regarding the production of an agricultural commodity [7]. Contract farming is already well-established in developed countries and regions [8]. Over 60 percent of large farms in the United States use contract farming, encompassing approximately 40 percent of the annual value of United States agricultural products. Contract farming has also substantially expanded in developing countries. According to the report on the operational trends and investment prospects forecast of China’s contract farming industry, the market size of China’s contract farming industry was about 62.2 billion yuan in 2019, occupying about 8% of the country’s total economy [9].

With the continuous economic development, contract farming has been widely adopted on a large scale. However, it has also begun to reveal some issues [10]. First, the compliance rate of contract farming is low. A study by Guo found that only 38 percent of enterprises or rural farm households in Jiangxi Province, China, complied with contract farming [11]. The low compliance rate can be attributed to various factors, such as an inadequate legal system [12], the high cost of prosecuting violators [13], and so on. Secondly, the average yield increase in contract farming is not sufficient to compensate for its high inputs. The study revealed that 39% of the farmers were engaged in contracts with elevated implied costs [14]. To address the aforementioned issues, providing subsidies for contract farming has become an important government initiative [15].

When the government implements contract farming subsidies, they can be categorized into two types based on the fiscal implementation: a single subsidy strategy and a combined subsidy strategy that incorporates multiple subsidy methods. The single subsidy strategy and the combined subsidy strategy have different impacts on social welfare, fiscal expenditure, and other related aspects. Due to the current economic environment of slowed or stagnant growth, government budgets are typically limited, and implementing combined strategies would result in excessive fiscal burdens [16]. Additionally, compared to combined subsidies, single subsidies are easier to implement and more widely used. Therefore, this paper only examines the single subsidy type.

Currently, various forms of the single subsidy strategy exist in contract farming, such as price subsidy, area subsidy, agricultural risk insurance subsidies and so on [2, 5]. Different government subsidy strategies have varying effects on the optimal decisions within the contract farming supply chain. The choice of subsidy strategy depends on the government’s desired approach to involvement in contract farming and the intended outcomes of the subsidy policy. In the context of contract farming under e-commerce assistance to farmers, the government aims to maximize social welfare by stimulating farmers’ production enthusiasm. Price subsidies and area subsidies can be viewed as implicit government guarantee mechanisms that reduce farmers’ production costs and encourage greater production [5]. At this point, these two subsidy methods align most closely with the government’s expectations. Given this, this paper only examines price subsidy models with different subsidy targets and area subsidy models.

However, in the cause of assisting farmers, relying solely on government subsidies can result in a shortage of marketing channels. In Zhaojue County, Sichuan Province, China, the government invested 1 million yuan in a poverty alleviation project to raise sheep. Still, the sheep could not be traded in the market due to lacking marketing channels [17]. It goes against the government’s initial intentions. Therefore, to integrate government policies with market mechanisms, both the government and enterprises must participate together. With the rapid development of e-commerce, introducing e-commerce platforms into contract farming has become increasingly popular, gradually forming the e-commerce assistance to farmers model. The so-called e-commerce assistance to farmers refers to a model where farmers use e-commerce platforms to sell agricultural products directly or indirectly to consumers, thereby promoting the sale of agricultural products and increasing farmers’ incomes [18, 19]. This model can serve as a bridge for the sale of agricultural products and help farmers improve production efficiency and economic benefits through various means such as technical support and market promotion, thereby alleviating rural poverty [20, 21].

Based on the background hereinbefore, the paper constructs a contract farming supply chain model composed of a single e-commerce platform and a single farmer. It investigates the optimal strategies for the contract farming supply chain under different government subsidy modes in the context of e-commerce assistance to farmers, as well as the impact of government subsidies and e-commerce assistance to farmers on the supply chain. Through this research, it aims to answer the following questions:

1. How do consumer preference for supporting farmers and consumer premiums influence the decisions and profits of the farmer and the e-commerce platform, and what impact do they have on total agricultural productions and social welfare?
2. How do government subsidies and output volatility impact on the decision-making of the farmer and the e-commerce platform? How do government subsidies affect the profits of the farmer and the e-commerce platform, as well as social welfare?
3. For the government, how should the most appropriate subsidy model be chosen from the perspectives of farm size, the purchase price of agricultural products, and profits of the farmer and the e-commerce platform?

**Fig 1:**
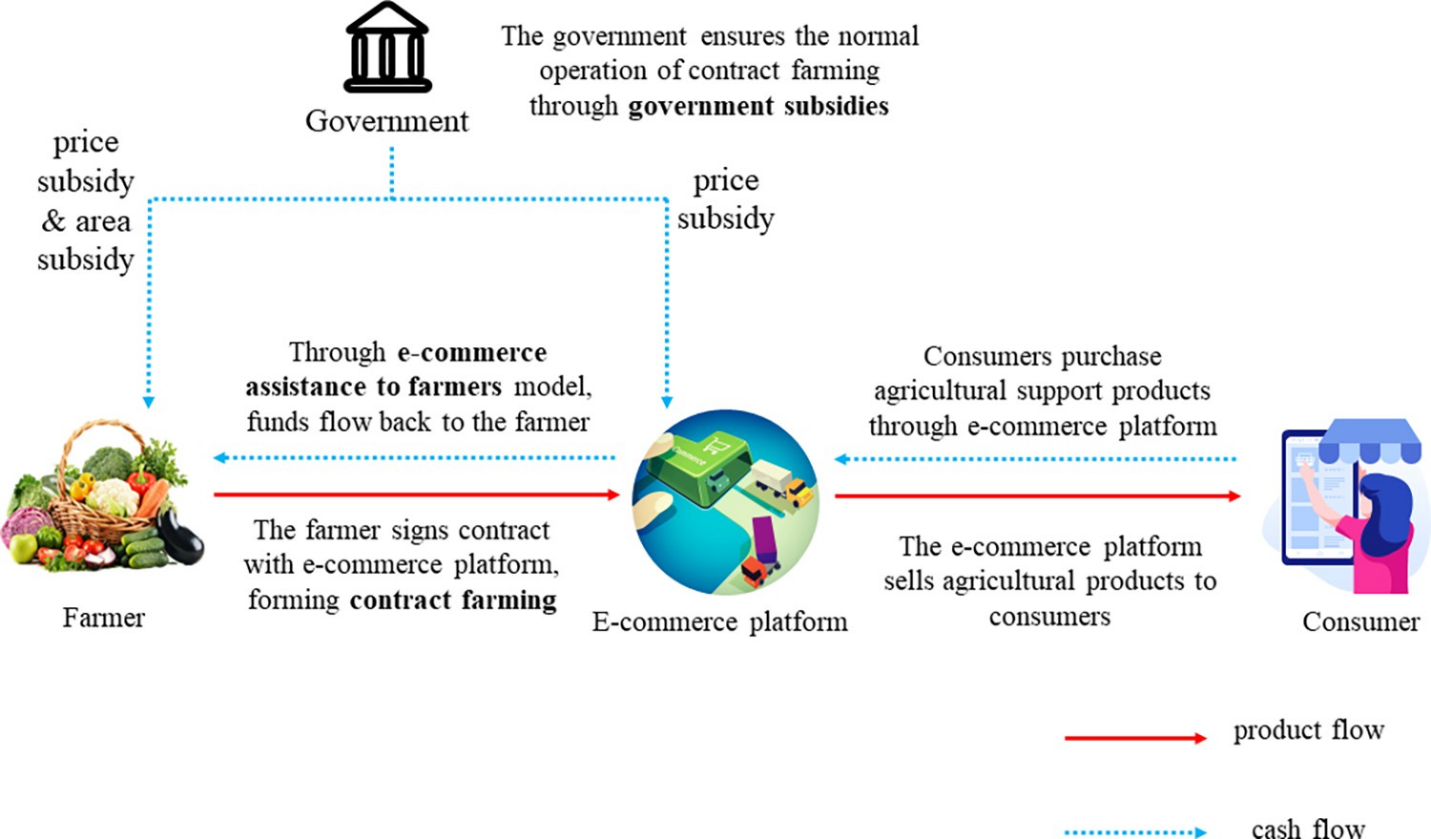
Graphical abstract for the paper.

This paper provides a graphical abstract to better illustrate the relationships between key concepts in the paper. The graphical abstract is shown in Fig 1.

### 2. Literature review

The concepts and methods proposed in this paper draw inspiration from various studies in previous literature. The literature review is structured around four primary themes: contract farming, government subsidies, e-commerce assistance to farmers, and the position of this study. The subsequent section provides a concise overview of these scholarly investigations.

### 2.1 Contract farming

Contract farming refers to an agricultural production and marketing model in which farmers organize and arrange production according to a contract signed with agricultural product buyers [22]. The current research on contract farming mainly focuses on three aspects. The first is the study of the organizational models of contract farming. In the “company + farmer” contract farming model, Kazaz and Webster [23] studied the impact of corporate risk preferences on this model. Pu [24] explored how to design the optimal contract in the “company + farmer” contract farming model. Based on the basic “company + farmer” model, some scholars have explored improved organizational models for contract farming. They introduced intermediary institutions between the company and farmers to enhance the bargaining power of farmers with the company. This evolved into organizational structures such as “company + cooperative + farmer” and “company + supplier + farmer”. Cui [25] developed a “company + cooperative + farmer” three-level contract farming supply chain model, exploring the optimization and decision-making of the supply chain under the Conditional Value-at-Risk (CVaR) criterion. Anderson and Monjardino [26] studied the optimal decisions of companies and suppliers in the “company + supplier + farmer” contract farming model. The second aspect is the analysis of contract design in contract farming based on the contract organizational model. During the contract design process, it typically focuses on contract acquisition quantity, market demand, and supply chain coordination. Wang’s research [27] found that retailers should sign contracts with farmers for lower production quantities to ensure that farmers can fulfill the contracts. After the harvest, supplementary contracts should be signed with farmers based on the actual situation. Hong’s research [20] found that companies should share positive market demand information with farmers when drafting contracts while concealing negative information. Ye’s research [28] found that the contract farming process should reasonably utilize mechanisms such as revenue sharing, production cost sharing, and deposit mechanisms. The third aspect is the positive benefits of contract farming. Existing research has found that contract farming can improve supply chain efficiency [29], reduce farmers’ production risks [28], and increase the commercialization and intensification of agriculture productions [30]. Consequently, it helps farmers increase production and income, enhances their welfare, and provides a basic guarantee for their sustainable operations [31, 32].

However, in the research on the aforementioned contract farming supply chain, few studies have examined e-commerce assistance to farmers, government subsidies, and contract farming within the same framework. This paper, based on the background of e-commerce assistance to farmers, investigates the decision-making in the contract farming supply chain under different government subsidy models.

### 2.2 Government subsidies

In the context of contract farming, government agricultural subsidies can promote agricultural production, stimulate the stable operation of the rural economy, and ensure national food security [28, 33]. In view of this, government agricultural subsidies have become a hot topic of research among scholars. Currently, government agricultural subsidies come in various forms, including subsidies based on the cost of production materials [34, 35], subsidies on the purchase price or purchase volume of agricultural products [36], and price loss subsidies [2]. It is noteworthy that supply chain decisions vary under different government agricultural subsidies. Therefore, the operation of supply chains under different government agricultural subsidies has become a topic of interest for scholars. Guda et al. [37] solved the equilibrium strategies for farmers and the government under a target price subsidy for agricultural products by establishing a game model. Chintapalli and Tang [38] argued that single government subsidies are not entirely beneficial to farmers and that the government needs to design and implement targeted subsidy policies. Consequently, some scholars have begun to discuss the scenario where the government provides multiple subsidy policies. Chen studied how the government should choose between input-oriented and output-oriented subsidy policies under conditions of uncertain output, cost, and price [39]. Xie [40] constructed a Stackelberg game model to seek the optimal decisions for each member of the supply chain under both target price subsidy and acreage subsidy mechanisms. Fu’s research [41] examined the different impacts of three subsidy policies: no subsidy strategy, fixed subsidy strategy, and agricultural risk coverage strategy, finding that the agricultural risk coverage subsidy policy significantly outperforms the no subsidy strategy and fixed subsidy strategy in encouraging farmers to invest in environmental sustainability under relatively harsh weather conditions. Zhang [42] explored a hybrid subsidy model combining output subsidies and environmental innovation subsidies, and investigated how to achieve optimal coordination in the supply chain.

Based on the operation of supply chains under different government agricultural subsidies, some scholars have analyzed the effects of implementing government agricultural subsidies and their impact on supply chains. Existing literature has analyzed various aspects such as farmers’ output and income, the stability of agricultural product market prices, agricultural enterprises’ profits, the prevalence of contract farming, and national food security [2, 39, 43]. Alizamir and He [2, 43] found that appropriate government agricultural subsidies not only encourage farmers to increase production and income and protect agricultural products from the effects of low-market price fluctuations, but also enable agricultural enterprises to achieve higher profits. Oya [10]suggested that suitable government agricultural subsidies can effectively promote the prevalence of contract farming. For example, in Southern Africa, government-implemented input and output subsidies have significantly increased the prevalence of contract farming. Chen [39] noted that while implementing government agricultural subsidy policies, countries also help to protect national food security. It is important to note that government agricultural subsidies must be implemented within an appropriate range; if subsidies are too low or too high, they may produce negative effects. Subsidies that are too low may fail to bring positive benefits to agriculture [44], while excessive subsidies may affect production motivation and hinder economic development [37].

The existing literature predominantly examines government subsidies that directly target farmers. However, this paper innovatively analyzes different government subsidy models when the subsidy recipients are either e-commerce platforms or farmers, offering flexibility for the government to make optimal subsidy decisions. Moreover, existing research on contract farming subsidies often overlooks issues related to social welfare. This paper examines the impact of government subsidies on social welfare and explores the most suitable government subsidy decisions from multiple perspectives.

### 2.3 E-commerce assistance to farmers

Current research on e-commerce assistance to farmers primarily focuses on two aspects: the intrinsic mechanisms of e-commerce in supporting farmers and its impact on the supply chain. Firstly, regarding the intrinsic mechanisms of e-commerce in supporting farmers, Zhou et al. [45] studied how e-commerce can choose the optimal funding model for farmers’ production between crowdfunding and traditional methods. Sun [46] considered the e-commerce assistance model and established a two-tier agricultural product supply chain model involving farmers and e-commerce platforms, examining production and pricing strategies under agency selling and reselling models. Secondly, regarding the impact of e-commerce assistance to farmers on the supply chain, most existing research primarily explores the influence of e-commerce assistance on exogenous variables within the supply chain, such as consumer preferences for supporting farmers [5], e-commerce platform fairness concerns [47], agricultural product price fluctuations [48], and supply chain information management [49].

In the aforementioned literature, it can be observed that, in current research on e-commerce assistance to farmers, few studies consider supply chain decision-making under different government subsidy models. Moreover, there is limited research discussing e-commerce assistance to farmers from the consumer’s perspective. This paper examines the e-commerce assistance to farmers model through two parameters: consumer preferences for supporting farmers and consumer premiums. This approach provides valuable insights for studying e-commerce assistance to farmers from the consumer’s perspective.

### 2.4 The position of this study

Currently, scholars’ attentions are gradually shifting towards the application of government subsidies in contract farming. However, there is a lack of exploration of the impacts of different subsidies models on contract farming. Additionally, there is a relative lack of research on the integration of the e-commerce assistance to farmers model into contract farming. In conclusion, this paper develops a model for the supply chain of contract farming with government subsidies under the backdrop of e-commerce assistance to farmers. The differences between this paper and previous research are as follows. First, it introduces the e-commerce assistance to farmers model into contract farming and considers the impact of yield uncertainty. Second, it provides a more detailed comparative discussion of various government subsidies models, contrasting unsubsidized, price subsidy, and area subsidy in the context of contract farming. Finally, this paper examines the impact of government subsidies from a social welfare perspective. Table 1 summarizes the relevant research questions examined in previous studies and compares them with the present study.

**Table 1:** Comparison of the most pertinent prior studies with the current research.

	Yield uncertainty	e-commerce assistance to farmers	Price subsidy	Area subsidy	social welfare
Peng and Pang (2019)	√			√	
Sun and Peng (2022)	√	√			
Xie et al. (2023)	√		√	√	√
Hong et al. (2023)	√				√
Shi et al. (2021)	√		√		√
He et al. (2023)			√		√
Fu et al. (2023)	√		√		
Kang et al. (2023)		√	√		
This study	√	√	√	√	√

The remainder of the paper is structured as follows: Section 3 outlines the model and presents its assumptions. Subsequently, Section 4 establishes the benchmark model without government subsidies. In Section 5, a model encompassing three distinct types of government subsidies is introduced. Section 6 is dedicated to model analysis and numerical simulations. Finally, Section 7 provides the conclusions.

### 3. Model description and assumptions

**Fig 2:**
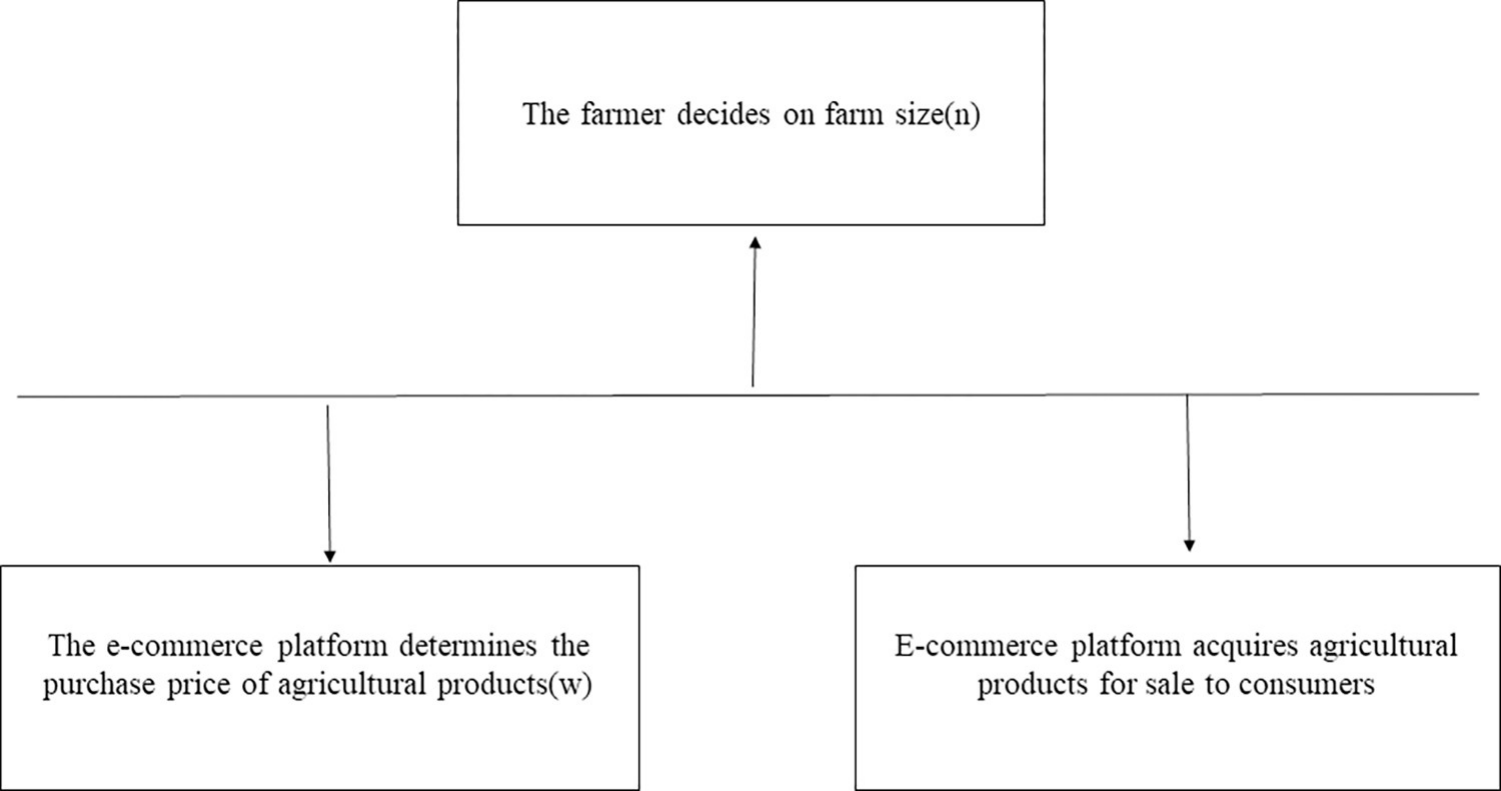
Supply chain decision sequence.

This paper considers a second-tier contract farming supply chain composed of an e-commerce platform and a farmer. The Stackelberg game model is proposed, with the e-commerce platform as the leader and the farmer as the follower in the supply chain. The decision sequence for the contract farming supply chain is as follows. In the first stage, the e-commerce platform promotes the purchase price of agricultural products w. In the second stage, the farmer decides on the farm size n to be planted. In the third stage, the e-commerce platform purchases the farming products and sells them to consumers (As shown in Fig 2).

To ensure the rationality of the research, the establishment of the model in this study is based on the following assumptions. In addition, the descriptions of notations involved in the model of this research are shown in Table 2.

**Table 2:** Research model symbols and descriptions.

Symbols	Explanation
p	The retail price of the agricultural product
w	Purchase price of agricultural products
y	Agricultural productions per unit area
n	Farm size
μ	Price elasticity coefficient
β	Percentage of government subsidies to the purchase price of agricultural products per unit planted in price subsidy, where β∈{0≤β≤1}
θ	Amount of government subsidies per acre of agricultural products planted in area subsidy
Q	Total production of agricultural products
c_1_	Farmer’s planting cost coefficient
φ	Consumer preference coefficient for supporting farmers, where φ∈{0≤φ≤1}
R_i_	Amount of government subsidies, where i∈{1,2}

1. The agricultural productions process is highly susceptible to various factors, such as adverse weather, pests, floods. Therefore, there is uncertainty in the agricultural productions yield. The actual unit area output per acre is yX. The agricultural productions per unit area is y and X is a positive continuous random variable, X>0, X follows the normal distribution. To facilitate further calculations, the following assumptions are made: E[X] = 1, var[X] = σ^2^ [5]. The total agricultural productions is expressed as: Q = ny [5], n is the farm size.
2. The farmer is assumed to be below the poverty standard [29, 50]. In addition, consumers have a preference for supporting farmers, and the coefficient of consumer preference for supporting farmers is φ. Consumers are willing to pay premiums for agricultural products produced by the farmer when the farmer’s income is below the poverty line standard. Define the premiums as Δπ [19], the Δπ range is: Δπ>0.
3. Assuming that the market inverse demand for agricultural products is linear. The inverse demand function is: p = α−μQX+φΔπ [5, 19, 46], where p, α and μ denote the retail price of the agricultural product, the market size and the price elasticity coefficient. Considering that the agricultural product market is close to a perfectly competitive market, it is assumed that the retail price of agricultural products p is exogenous [51].
4. Agricultural production often exhibits diseconomies of scale, so the production costs of farmers are typically represented non-linearly [51, 52]. The expression of farmer’s cost function is: C(Q) = c_1_Q^2^ [2], which c_1_ denotes the cost coefficient of farmer’s cultivation.
5. R_i_ is the government subsidies in different ways, R_1_ is the government subsidies by price, R_1_ = βwQX, β is the proportion of government subsidies per unit of the purchase price of agricultural products, R_2_ is the government subsidies by area, R_2_ = θn, θ is the amount of subsidies per acre of planted agricultural products [5, 40, 41].
6. Assuming that the consumer’s perceived value s_i_ for agricultural products are heterogeneous, the consumer’s perceived value s_i_ for agricultural products are uniformly distributed on [0,1], so the consumer surplus can be expressed as $\int_{\alpha -\mu nyX+\varphi \Delta \pi }^{\alpha }s_{i}[s_{i}(\alpha -\mu nyX+\varphi \Delta \pi )]ds_{i}$, which can be further simplified as $\frac{\mu }{2}Q^{2}X^{2}$ [2].

This study considers four different models for contract farming: (1) The benchmark model, without government subsidies, is denoted as NN. (2) The price subsidy targeting the farmer, which is denoted as PF. (3) The price subsidy targeting the e-commerce platform, which is denoted as PE. (4) The area subsidy model is denoted as AF. To facilitate the model description, the subscript F denotes the farmer and the subscript C denotes the e-commerce platform.

## 4. Benchmark model

Firstly, establish a benchmark model without government subsidies as a reference model for different subsidy schemes.

In the unsubsidized model, the farmer’s profit function consists of two parts: one part is the profit from selling agricultural products to e-commerce platform, and the other part is the production costs [5, 10], so the farmer’s profit expression is:

$$\pi_{F}^{NN}=wQX-c_{1}Q^{2}$$ (1)

The expected profit function of the farmer is:

$$E[\pi_{F}^{NN}]=nwy-n^{2}y^{2}c_{1}$$ (2)

The e-commerce platform profit function consists of two parts: one is the profit gained from the sale of agricultural products to consumers, and the other is the cost spent on the acquisition of agricultural products [34, 38]:

$$\pi_{C}^{NN}=(p-w)QX$$ (3)

The expected profit function of the e-commerce platform is:

$$E[\pi_{C}^{NN}]=-\frac{w(w\mu (1+\sigma^{4})+2(w-\alpha -\Delta \pi \varphi )c_{1})}{4c_{1}^{2}}$$ (4)

According to the backward induction, first, solve for the decision variable of farm size in farmer decision-making, and then solve for the decision variable of the purchase price of agricultural products for the e-commerce platform.


$${n^{NN}}^{*}=\frac{\alpha +\Delta \pi \varphi }{2y(\mu +\mu \sigma^{4}+2c_{1})}$$ (5)

$${w^{NN}}^{*}=\frac{(\alpha +\Delta \pi \varphi )c_{1}}{\mu +\mu \sigma^{4}+2c_{1}}$$ (6)

Substitute the equilibrium solution of decision variables into the profit function, thereby deriving the optimal profit functions for the farmer and the e-commerce platform under the unsubsidized mode.


$${\pi_{F}^{NN}}^{*}=\frac{{\left(\alpha +\Delta \pi \varphi \right)}^{2}c_{1}}{4{\left(\mu +\mu \sigma^{4}+2c_{1}\right)}^{2}}$$ (7)

$${\pi_{C}^{NN}}^{*}=\frac{{\left(\alpha +\Delta \pi \varphi \right)}^{2}}{4(\mu +\mu \sigma^{4}+2c_{1})}$$ (8)

Since there are no government subsidies, the social welfare in this case consists of three components: the farmer’s profit, the e-commerce platform’s profit, and the consumer surplus. The social welfare function is:

$${SW}^{NN}=\pi_{F}+\pi_{C}+\frac{\mu }{2}Q^{2}X^{2}$$ (9)

The expected social welfare function is:

$$E[{SW}^{NN}]=\frac{3{\left(\alpha +\Delta \pi \varphi \right)}^{2}}{8(\mu +\mu \sigma^{4}+2c_{1})}$$ (10)

## 5. Three types of subsidies

In reality, there are various forms of government subsidies for contract farming supply chains. This section constructs supply chain models under three subsidy schemes, which are: price subsidy the subsidy target is the farmer (section 5.1), price subsidy the subsidy target is the e-commerce platform (section 5.2), and area subsidy (section 5.3).

### 5.1 Price subsidy scenario with the farmer as the subsidy target

The price subsidy model where subsidy target is the farmer is tailored to farmer’s actual production levels, is formulated to alleviate the challenges they encounter in agricultural productions. Numerous real-world instances exemplify this strategy, including the direct subsidies provided to farmers in the European Union, with the goal of enhancing agricultural productions and fostering market harmonization for agricultural products [53].

When the government directly subsidizes the farmer in the form of price subsidy. The government subsidizes a percentage of the price per unit of agricultural productions. The amount of government subsidies at this time is expressed as R_1_. The expression for R_1_ is:

$$R_{1}=\beta wQX$$ (11)

The profit function of farmers undergoes certain modifications compared to the benchmark model. In such cases, the farmer’s profit comprises three components: the profit from selling agricultural products to e-commerce platform, the production costs, and the price subsidy for farmer. The farmer’s profit function becomes:

$${\pi_{F}}^{PF}=wQX+R_{1}-c_{1}Q^{2}$$ (12)

The expected profit function of the farmer can be expressed by the following equation:

$$E[{\pi_{F}}^{PF}]=ny(w+w\beta -nyc_{1})$$ (13)

The profit function of the e-commerce platform still consists of two parts: the profit gained from the sale of agricultural products, and the cost spent on the acquisition of agricultural products.


$${\pi_{C}}^{PF}=(p-w)QX$$ (14)

The expected profit function of the e-commerce platform is:

$$E[{\pi_{C}}^{PF}]=-\frac{w(1+\beta )(w(1+\beta )\mu (1+\sigma^{4})+2(w-\alpha -\Delta \pi \varphi )c_{1})}{4c_{1}^{2}}$$ (15)

Using backward induction, derive the equilibrium results of farm size and the purchase price of agricultural products under the model of price subsidy the subsidies target is the farmer.


$${n^{PF}}^{*}=\frac{\left(1+\beta )(\alpha +\Delta \pi \varphi \right)}{2y((1+\beta )\mu (1+\sigma^{4})+2c_{1})}$$ (16)

$${w^{PF}}^{*}=\frac{(\alpha +\Delta \pi \varphi )c_{1}}{\mu +\beta \mu +\mu \sigma^{4}+\beta \mu \sigma^{4}+2c_{1}}$$ (17)

The equilibrium solutions n^PF*^ and w^PF*^ are brought into the expected profit function of the farmer and the e-commerce platform to obtain the optimal profit functions of farmer and e-commerce platform.


$${{\pi_{F}}^{PF}}^{*}=\frac{{\left(1+\beta \right)}^{2}{\left(\alpha +\Delta \pi \varphi \right)}^{2}c_{1}}{4{\left((1+\beta )\mu (1+\sigma^{4})+2c_{1}\right)}^{2}}$$ (18)

$${{\pi_{C}}^{PF}}^{*}=\frac{(1+\beta ){\left(\alpha +\Delta \pi \varphi \right)}^{2}}{4(1+\beta )\mu (1+\sigma^{4})+8c_{1}}$$ (19)

There is a distinction between social welfare under the price subsidy model targeting the farmer and the benchmark model. In the current model, the social welfare function comprises four components: farmer profit, e-commerce platform profit, consumer surplus, and government subsidies. The expression for social welfare is as follows.


$${SW}^{PF}=\pi_{F}+\pi_{C}-R_{1}+\frac{1}{2}\mu Q^{2}X^{2}$$ (20)

The expected social welfare is:

$$E[{SW}^{PF}]=\frac{\left(1+\beta ){\left(\alpha +\Delta \pi \varphi \right)}^{2}(3(1+\beta )\mu (1+\sigma^{4})-2(-3+\beta )c_{1}\right)}{8{\left((1+\beta )\mu (1+\sigma^{4})+2c_{1}\right)}^{2}}$$ (21)

In the presence of government subsidies, some interesting properties are discerned.

**Property 1:**

$$\frac{\partial {n^{PF}}^{*}}{\partial \beta }=\frac{(\alpha +\Delta \pi \varphi )c_{1}}{y{\left((1+\beta )\mu (1+\sigma^{4})+2c_{1}\right)}^{2}}>0,\frac{\partial {w^{PF}}^{*}}{\partial \beta }=-\frac{\mu (1+\sigma^{4})(\alpha +\Delta \pi \varphi )c_{1}}{{\left((1+\beta )\mu (1+\sigma^{4})+2c_{1}\right)}^{2}}<0$$

$$\frac{\partial {{\pi_{F}}^{PF}}^{*}}{\partial \beta }=\frac{(1+\beta ){\left(\alpha +\Delta \pi \varphi \right)}^{2}c_{1}^{2}}{{\left((1+\beta )\mu (1+\sigma^{4})+2c_{1}\right)}^{3}}>0,\frac{\partial {{\pi_{C}}^{PF}}^{*}}{\partial \beta }=\frac{{\left(\alpha +\Delta \pi \varphi \right)}^{2}c_{1}}{2{\left((1+\beta )\mu (1+\sigma^{4})+2c_{1}\right)}^{2}}>0$$

Property 1 shows that as government subsidies increase, farm size, farmer and e-commerce platform profit all rise, while the purchase price of agricultural products decreases. This is because the more subsidies the government provides to farmers, the lower the costs they bear, which in turn incentivizes them to expand farm size and increase profits. The reason of the purchase price of agricultural products decreases as government subsidies increase is that farmers’ incomes rise and their financial burdens lighten with the added subsidies. As a result, farmers can accept lower purchase prices for their products, allowing e-commerce platforms to reduce the purchase prices for agricultural products. Consequently, the profits of e-commerce platforms also increase.

### 5.2 Price subsidy scenario with the e-commerce platform as the subsidy target

To enhance the enthusiasm of e-commerce platforms in supporting farmers, the government provides subsidies to e-commerce platforms to stimulate the acquisition and sale of supportive agricultural products. For instance, in 2018, Bangladesh allocated a substantial sum of Tk 44.81 billion to subsidize e-commerce companies that ventured into rural areas to procure agricultural products [54].

The form of government subsidies is the same as in model 5.1, with the subsidies recipient replaced by an e-commerce platform.


$$R_{1}=\beta wQX$$ (22)

The farmer’s profit function is similar to the benchmark model, with the farmer’s decision variable remaining at farm size. The profit function is modeled as follows:

$${\pi_{F}}^{PE}=wQX-c_{1}Q^{2}$$ (23)

The farmer’s expected profit expression is:

$$E[{\pi_{F}}^{PE}]=nwy-n^{2}y^{2}c_{1}$$ (24)

The government subsidizes e-commerce platforms in the form of price subsidy, the e-commerce platform’s profit function is changed. On the basis of the above model, the government subsidies component has been added. The profit function of the e-commerce platform is as follows:

$${\pi_{C}}^{PE}=(p-w)QX+R_{1}$$ (25)

The expected profit function of the e-commerce platform is:

$$E[{\pi_{C}}^{PE}]=-\frac{w(w\mu (1+\sigma^{4})-2(\alpha +w(-1+\beta )+\Delta \pi \varphi )c_{1})}{4c_{1}^{2}}$$ (26)

Using backward induction, derive the equilibrium results of the supply chain operation in the model with price subsidy targeted at e-commerce platforms.


$${n^{PE}}^{*}=\frac{\alpha +\Delta \pi \varphi }{2y(\mu +\mu \sigma^{4}+2c_{1}-2\beta c_{1})}$$ (27)

$${w^{PE}}^{*}=\frac{(\alpha +\Delta \pi \varphi )c_{1}}{\mu +\mu \sigma^{4}+2c_{1}-2\beta c_{1}}$$ (28)

Bringing in the equilibrium solutions n^PE*^ and w^PE*^, the optimal returns of the farmer and the e-commerce platform can be obtained as:

$${{\pi_{F}}^{PE}}^{*}=\frac{{\left(\alpha +\Delta \pi \varphi \right)}^{2}c_{1}}{4{\left(\mu (1+\sigma^{4})-2(-1+\beta )c_{1}\right)}^{2}}$$ (29)

$${{\pi_{C}}^{PE}}^{*}=\frac{{\left(\alpha +\Delta \pi \varphi \right)}^{2}}{4\mu (1+\sigma^{4})-8(-1+\beta )c_{1}}$$ (30)

The social welfare under the price subsidy for the e-commerce platform is consistent with that under the price subsidy for the farmer model.


$${SW}^{PE}=\frac{{\left(\alpha +\Delta \pi \varphi \right)}^{2}(\mu (2+X^{2}+2\sigma^{4})+(6-4(1+X)\beta )c_{1})}{8{\left(\mu (1+\sigma^{4})-2(-1+\beta )c_{1}\right)}^{2}}$$ (31)

The expected social welfare function is:

$$E[{SW}^{PE}]=\frac{{\left(\alpha +\Delta \pi \varphi \right)}^{2}(3\mu (1+\sigma^{4})+(6-8\beta )c_{1})}{8{\left(\mu (1+\sigma^{4})-2(-1+\beta )c_{1}\right)}^{2}}$$ (32)

There are also some interesting properties to the model where the price subsidy is subsidized by e-commerce platform.

**Property 2:**

$$\frac{\partial {w^{PE}}^{*}}{\partial \beta }=\frac{2(\alpha +\Delta \pi \varphi )c_{1}^{2}}{{\left(\mu +\mu \sigma^{4}+2c_{1}-2\beta c_{1}\right)}^{2}}>0,\frac{\partial {n^{PE}}^{*}}{\partial \beta }=\frac{(\alpha +\Delta \pi \varphi )c_{1}}{y{\left(\mu +\mu \sigma^{4}+2c_{1}-2\beta c_{1}\right)}^{2}}>0$$

$$\frac{\partial {{\pi_{F}}^{PE}}^{*}}{\partial \beta }=\frac{{\left(\alpha +\Delta \pi \varphi \right)}^{2}c_{1}^{2}}{{\left(\mu (1+\sigma^{4})-2(-1+\beta )c_{1}\right)}^{3}}>0,\frac{\partial {{\pi_{C}}^{PE}}^{*}}{\partial \beta }=\frac{8{\left(\alpha +\Delta \pi \varphi \right)}^{2}c_{1}}{{\left(4\mu (1+\sigma^{4})-8(-1+\beta )c_{1}\right)}^{2}}>0$$

From Property 2, it becomes evident that when the government enhances its subsidies to the e-commerce platform, this will lead to an increase in the purchase price of agricultural products an expansion of the farm size and higher profits for both the farmer and the e-commerce platform. This occurs because when the government increases the subsidy rate for e-commerce platforms, the e-commerce platform is able to accept higher than usual purchase price of agricultural products, and by raising the purchase price of agricultural products, the e-commerce platform in turn shares the cost of growing agricultural products with the farmer. As the purchase price of agricultural products increases, farmers become more profitable and have an incentive to allocate more resources to agriculture, thereby expanding their farm size. When farmers expand their farm size, e-commerce platforms can obtain more agricultural products. Combined with government subsidies, the profits of e-commerce platforms will also increase.

### 5.3 Area subsidy scenario

The price subsidy scenarios discussed above exist challenges in certain situations. For instance, they may be difficult to thoroughly supervise, leading to potential discrepancies in the authenticity of subsidy amounts [5]. The area subsidy scenario is more intuitive and less likely to result in false subsidy amounts. Moreover, the starting point of price subsidy and area subsidy is very different, the price subsidy focus on maintaining the price stability of agricultural products, while the essence of area subsidy is to encourage the production of agricultural products to ensure the stability of planting area and yield. The overarching objective of area subsidy is to encourage adjustments in planting structures, optimize the agricultural planting industry, and enhance the effectiveness of emerging agrarian policies. For instance, the provincial government of Guangxi, China, implemented a policy that provides a subsidy of 100 yuan per mu of cultivated land, thus reducing the production-related pressures on farmers and fostering increased enthusiasm for production improvements [39]. The following section discusses area subsidy.

The government gives farmers a fixed number of subsidies per acre based on acreage. The amount of government subsidies by area is denoted as R_2_. The expression for R_2_ is:

$$R_{2}=\theta n$$ (33)

The government directly subsidizes the farmer according to the area subsidy. The profits function of the farmer increases the government subsidies part based on the benchmark model, so the profit function of the farmer is:

$${\pi_{F}}^{AF}=wQX-c_{1}Q^{2}+R_{2}$$ (34)

The expected profit function of a farmer in this model is:

$$E[{\pi_{F}}^{AF}]=nwy+n\theta -n^{2}y^{2}c_{1}$$ (35)

The e-commerce platform profit function is similar to the benchmark model, and the expression for the e-commerce platform profit function is:

$${\pi_{C}}^{AF}=(p-w)QX$$ (36)

The expected profit function of the e-commerce platform is:

$$E[{\pi_{C}}^{AF}]=-\frac{\left(wy+\theta )((wy+\theta )\mu (1+\sigma^{4})+2y(w-\alpha -\Delta \pi \varphi )c_{1}\right)}{4y^{2}c_{1}^{2}}$$ (37)

Using backward induction, derive the equilibrium results of the supply chain operation in the model with area subsidy.


$${n^{AF}}^{*}=\frac{\theta +y(\alpha +\Delta \pi \varphi )}{2y^{2}(\mu +\mu \sigma^{4}+2c_{1})}$$ (38)

$${w^{AF}}^{*}=\frac{-\theta \mu -\theta \mu \sigma^{4}+y\alpha c_{1}-\theta c_{1}+y\Delta \pi \varphi c_{1}}{y(\mu +\mu \sigma^{4}+2c_{1})}$$ (39)

The equilibrium solutions n^AF*^ and w^AF*^ are brought into the profit functions of the farmer and the e-commerce platform, respectively, to obtain the optimal profits of the farmer and the e-commerce platform, denoted by π_F_^AF*^ and π_C_^AF*^.


$${{\pi_{F}}^{AF}}^{*}=\frac{{\left(\theta +y(\alpha +\Delta \pi \varphi )\right)}^{2}c_{1}}{4y^{2}{\left(\mu +\mu \sigma^{4}+2c_{1}\right)}^{2}}$$ (40)

$${{\pi_{C}}^{AF}}^{*}=\frac{{\left(\theta +y(\alpha +\Delta \pi \varphi )\right)}^{2}}{4y^{2}(\mu +\mu \sigma^{4}+2c_{1})}$$ (41)

The social welfare structure under the area subsidy model aligns with the above. The social welfare function is outlined below:

$${SW}^{AF}=\frac{3y^{2}\alpha^{2}+2y\alpha \theta -\theta^{2}+6y^{2}\alpha \Delta \pi \varphi +2y\Delta \pi \theta \varphi +3y^{2}{\Delta \pi }^{2}\varphi^{2}}{8y^{2}(\mu +\mu \sigma^{4}+2c_{1})}$$ (42)

The expected social welfare function under the area subsidy model is:

$$E[{SW}^{AF}]=\frac{-\theta^{2}+2y\theta (\alpha +\Delta \pi \varphi )+3y^{2}{\left(\alpha +\Delta \pi \varphi \right)}^{2}}{8y^{2}(\mu +\mu \sigma^{4}+2c_{1})}$$ (43)

The Property under the area subsidy model is broadly similar to the previous model but with some differences, as follows:

**Property 3:**

$$\frac{\partial {n^{AF}}^{*}}{\partial \theta }=\frac{1}{2y^{2}(\mu +\mu \sigma^{4}+2c_{1})}>0,\frac{\partial {w^{AF}}^{*}}{\partial \theta }=-\frac{\mu +\mu \sigma^{4}+c_{1}}{y\mu +y\mu \sigma^{4}+2yc_{1}}<0$$

$$\frac{\partial {{\pi_{F}}^{AF}}^{*}}{\partial \theta }=\frac{(\theta +y(\alpha +\Delta \pi \varphi ))c_{1}}{2y^{2}{\left(\mu +\mu \sigma^{4}+2c_{1}\right)}^{2}}>0,\frac{\partial {{\pi_{C}}^{AF}}^{*}}{\partial \theta }=\frac{\theta +y(\alpha +\Delta \pi \varphi )}{2y^{2}(\mu +\mu \sigma^{4}+2c_{1})}>0$$

From Property 3, we can see that, as the area subsidy rate increases, farm sizes expand, purchase prices of agricultural products decrease, and both farmers and e-commerce platforms benefit. Peng’s research similarly found that government subsidies are positively correlated with farm size and the profits of enterprises and farmers, and negatively correlated with the purchase prices of agricultural products [5]. However, Peng’s study did not consider the e-commerce assistance to farmers model. The cause of Property 3 is: government subsidies based on farm size incentivize the farmer to continually expand their farm size to receive more subsidies, which reduces their production costs and allows them to accept lower purchase prices. E-commerce platforms, seeking profit, will then lower their purchase prices. As a result, both parties benefit from the increased farm size and reduced purchase prices.

## 6. Sensitivity analysis, comparative analysis and numerical results

In this section, a sensitivity analysis was conducted for the key parameters of consumer preference for supporting farmers and consumer payment premiums in the context of the e-commerce assistance model. Additionally, a comparative analysis was performed for farm size, the purchase price of agricultural products, and profits for farmers and e-commerce platforms under different subsidy models. Finally, numerical simulations were carried out for all the analyzed content.

### 6.1 Sensitivity analysis

**Proposition 1:**

$$\frac{\partial {n^{NN}}^{*}}{\partial \sigma }=-\frac{2\mu \sigma^{3}(\alpha +\Delta \pi \varphi )}{y{\left(\mu +\mu \sigma^{4}+2c_{1}\right)}^{2}}<0,\frac{\partial {n^{PF}}^{*}}{\partial \sigma }=-\frac{2{\left(1+\beta \right)}^{2}\mu \sigma^{3}(\alpha +\Delta \pi \varphi )}{y{\left((1+\beta )\mu (1+\sigma^{4})+2c_{1}\right)}^{2}}<0$$

$$\frac{\partial {n^{PE}}^{*}}{\partial \sigma }=-\frac{2\mu \sigma^{3}(\alpha +\Delta \pi \varphi )}{y{\left(\mu +\mu \sigma^{4}+2c_{1}-2\beta c_{1}\right)}^{2}}<0,\frac{\partial {n^{AF}}^{*}}{\partial \sigma }=-\frac{2\mu \sigma^{3}(\theta +y(\alpha +\Delta \pi \varphi ))}{y^{2}{\left(\mu +\mu \sigma^{4}+2c_{1}\right)}^{2}}<0$$

$$\frac{\partial {w^{NN}}^{*}}{\partial \sigma }=-\frac{4\mu \sigma^{3}(\alpha +\Delta \pi \varphi )c_{1}}{{\left(\mu +\mu \sigma^{4}+2c_{1}\right)}^{2}}<0,\frac{\partial {w^{PF}}^{*}}{\partial \sigma }=-\frac{4(1+\beta )\mu \sigma^{3}(\alpha +\Delta \pi \varphi )c_{1}}{{\left((1+\beta )\mu (1+\sigma^{4})+2c_{1}\right)}^{2}}<0$$

$$\frac{\partial {w^{PE}}^{*}}{\partial \sigma }=-\frac{4\mu \sigma^{3}(\alpha +\Delta \pi \varphi )c_{1}}{{\left(\mu +\mu \sigma^{4}+2c_{1}-2\beta c_{1}\right)}^{2}}<0,\frac{\partial {w^{AF}}^{*}}{\partial \sigma }=-\frac{4\mu \sigma^{3}(\theta +y(\alpha +\Delta \pi \varphi ))c_{1}}{y{\left(\mu +\mu \sigma^{4}+2c_{1}\right)}^{2}}<0$$

**Fig 3:**
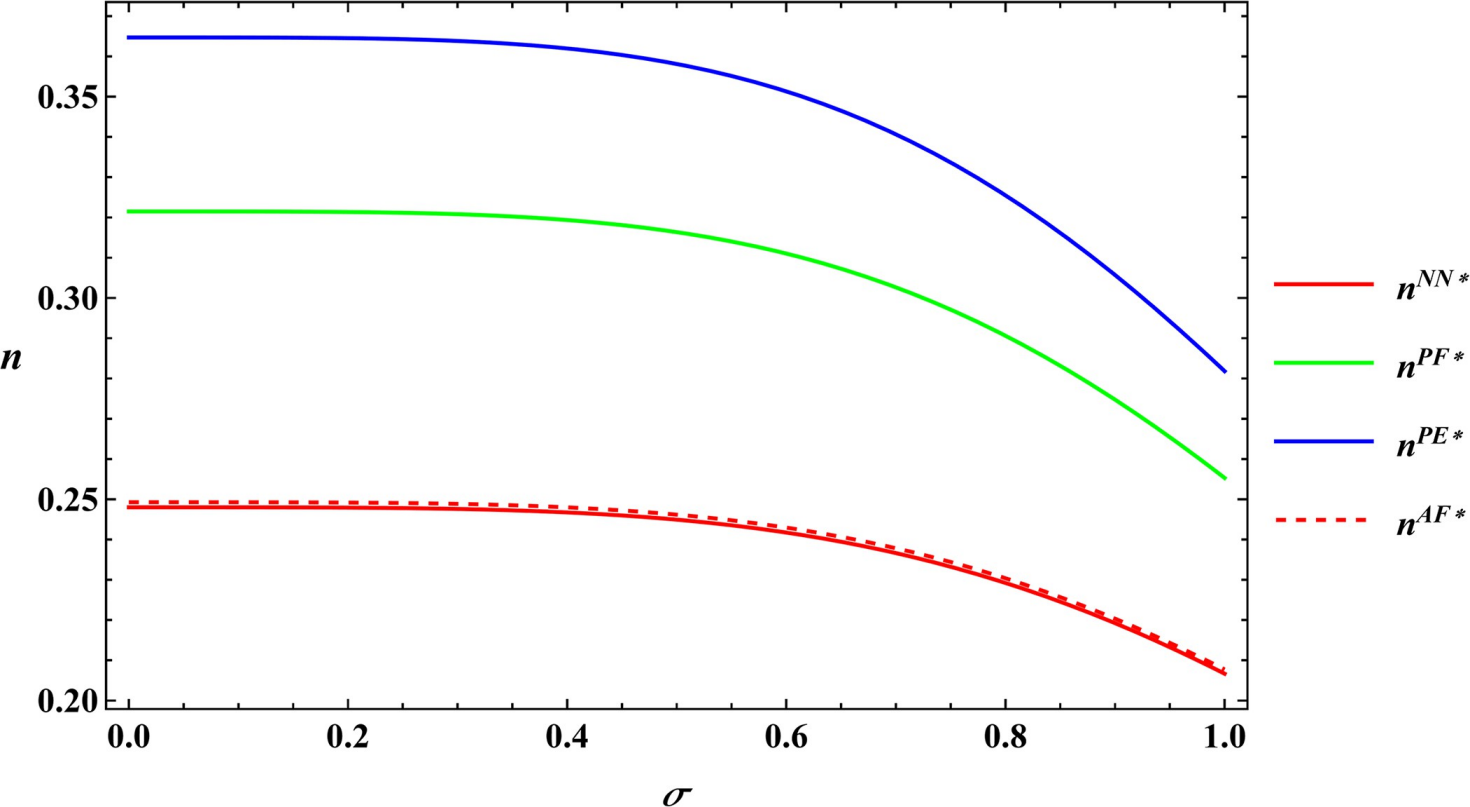
Sensitivity analysis of output volatility in the farm size.

**Fig 4:**
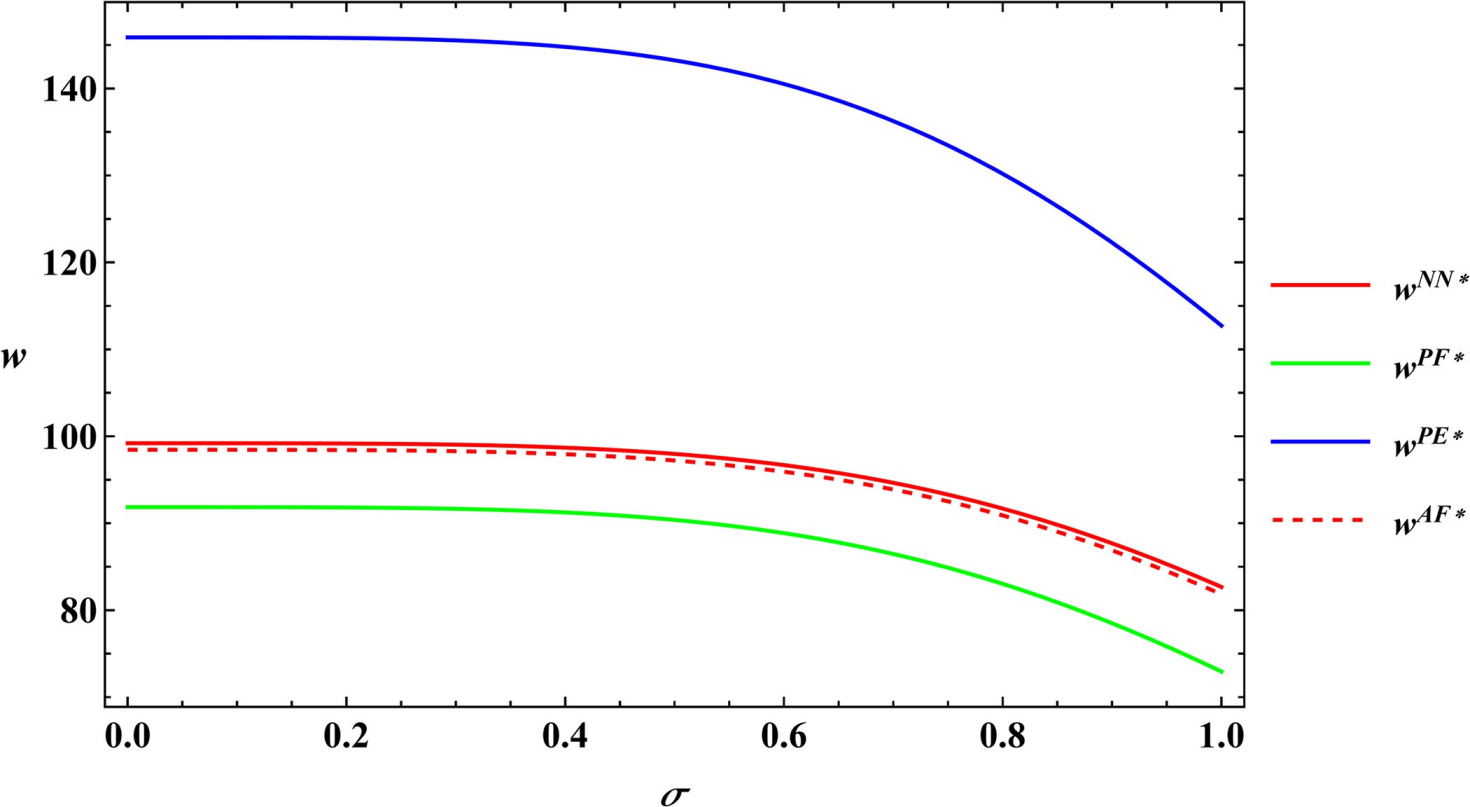
Sensitivity analysis of output volatility in the purchase price of agricultural products.

The following text uses numerical simulations to more clearly illustrate Proposition 1. The parameter values utilized in these simulations are adopted from Peng’s literature [5], with the specific parameter values outlined below. α = 8, μ = 5, c_1_ = 10, φ = 0.08, β = 0.4, Δπ = 3000, y = 20, θ = 25. The numerical simulation results are plotted as Figs 3 and 4.

The numerical simulation substantiates Proposition 1. Building upon this conclusion and the insights gained from the numerical simulation. It can be concluded that irrespective of the government subsidy forms, farm size and the purchase price of agricultural products decrease as output volatility increases. Peng’s research explored the impact of output volatility under the area subsidy model [5]. The results showed that output volatility is negatively correlated with both farm size and the purchase price of agricultural products, which is consistent with the conclusions of this paper. Building on Peng’s research, this paper compares the impact of output volatility under four subsidy models: no subsidy, price subsidy with the farmer as the subsidy target, price subsidy with the e-commerce platform as the subsidy target and area subsidy. It finds that the impact of output volatility under the no subsidy and price subsidy models is the same as under the area subsidy model. The phenomenon described in Proposition 1 occurs because when output fluctuations are significant, risk-averse farmers tend to reduce the farm size. Simultaneously, e-commerce platforms must contend with a more volatile output of agricultural products and increased market competition, compelling them to mitigate risk by lowering their purchasing prices. This creates a vicious circle in which lower purchase price of agricultural products in turn weaken farmers’ incentives to produce and cause them to continually downsize their farm size.

**Proposition 2:**

$$\frac{\partial {n^{NN}}^{*}}{\partial \varphi }=\frac{\Delta \pi }{2y(\mu +\mu \sigma^{4}+2c_{1})}>0,\frac{\partial {n^{PF}}^{*}}{\partial \varphi }=\frac{(1+\beta )\Delta \pi }{2y((1+\beta )\mu (1+\sigma^{4})+2c_{1})}>0$$

$$\frac{\partial {n^{PE}}^{*}}{\partial \varphi }=\frac{\Delta \pi }{2y(\mu (1+\sigma^{4})-2(-1+\beta )c_{1})}>0,\frac{\partial {n^{AF}}^{*}}{\partial \varphi }=\frac{\Delta \pi }{2y(\mu +\mu \sigma^{4}+2c_{1})}>0$$

$$\frac{\partial {n^{NN}}^{*}}{\partial \Delta \pi }=\frac{\varphi }{2y(\mu +\mu \sigma^{4}+2c_{1})}>0,\frac{\partial {n^{PF}}^{*}}{\partial \Delta \pi }=\frac{(1+\beta )\varphi }{2y((1+\beta )\mu (1+\sigma^{4})+2c_{1})}>0$$

$$\frac{\partial {n^{PE}}^{*}}{\partial \Delta \pi }=\frac{\varphi }{2y(\mu +\mu \sigma^{4}+2c_{1}-2\beta c_{1})}>0,\frac{\partial {n^{AF}}^{*}}{\partial \Delta \pi }=\frac{\varphi }{2y(\mu +\mu \sigma^{4}+2c_{1})}>0$$

$$\frac{\partial {w^{NN}}^{*}}{\partial \varphi }=\frac{\Delta \pi c_{1}}{\mu +\mu \sigma^{4}+2c_{1}}>0,\frac{\partial {w^{PF}}^{*}}{\partial \varphi }=\frac{\Delta \pi c_{1}}{(1+\beta )\mu (1+\sigma^{4})+2c_{1}}>0$$

$$\frac{\partial {w^{PE}}^{*}}{\partial \varphi }=\frac{\Delta \pi c_{1}}{\mu +\mu \sigma^{4}+2c_{1}-2\beta c_{1}}>0,\frac{\partial {w^{AF}}^{*}}{\partial \varphi }=\frac{\Delta \pi c_{1}}{\mu +\mu \sigma^{4}+2c_{1}}>0$$

$$\frac{\partial {w^{NN}}^{*}}{\partial \Delta \pi }=\frac{\varphi c_{1}}{\mu +\mu \sigma^{4}+2c_{1}}>0,\frac{\partial {w^{PF}}^{*}}{\partial \Delta \pi }=\frac{\varphi c_{1}}{(1+\beta )\mu (1+\sigma^{4})+2c_{1}}>0$$

$$\frac{\partial {w^{PE}}^{*}}{\partial \Delta \pi }=\frac{\varphi c_{1}}{\mu +\mu \sigma^{4}+2c_{1}-2\beta c_{1}}>0,\frac{\partial {w^{AF}}^{*}}{\partial \Delta \pi }=\frac{\varphi c_{1}}{\mu +\mu \sigma^{4}+2c_{1}}>0$$

**Fig 5:**
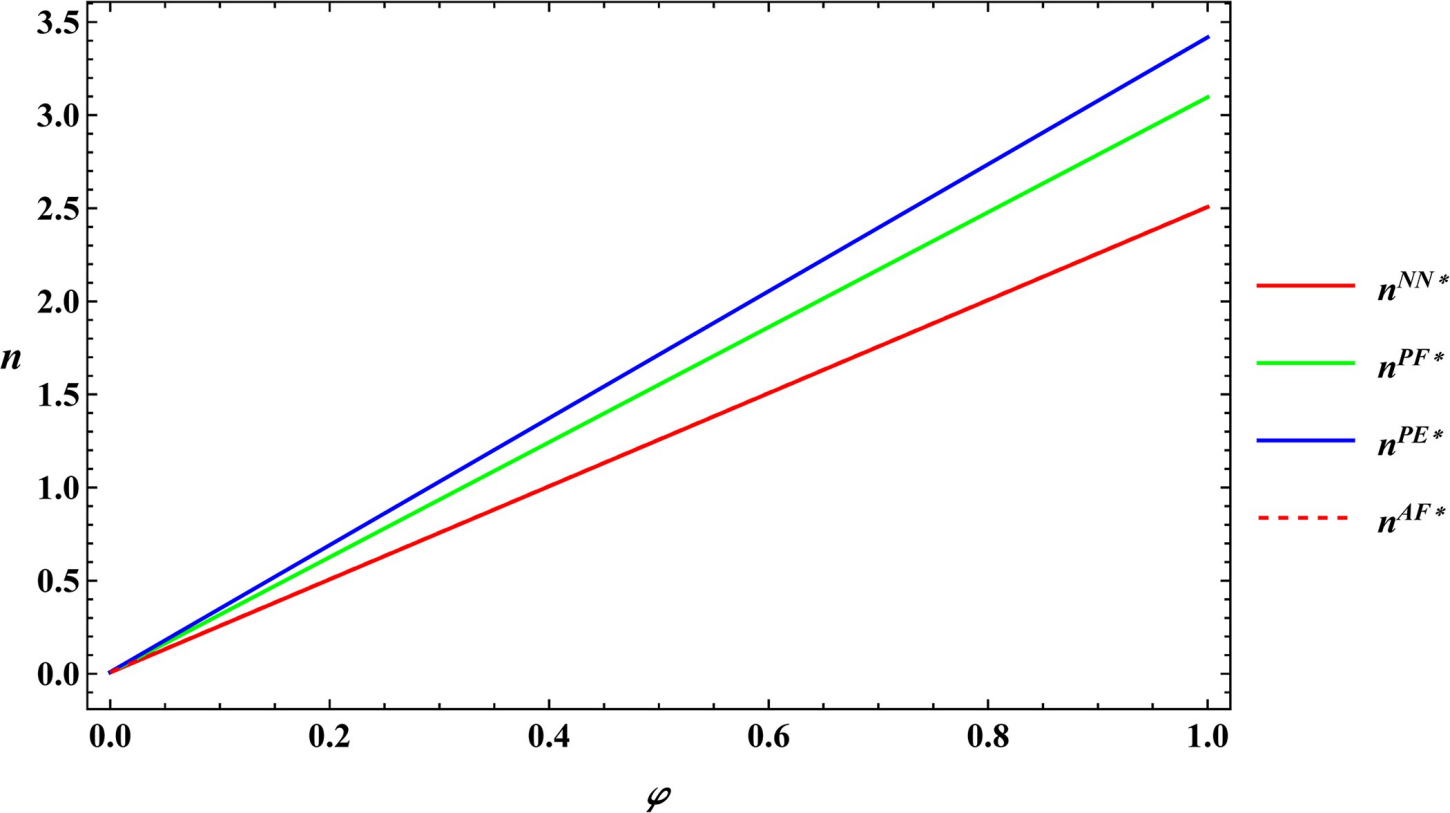
Sensitivity analysis of consumer preference for supporting farmers in farm size.

**Fig 6:**
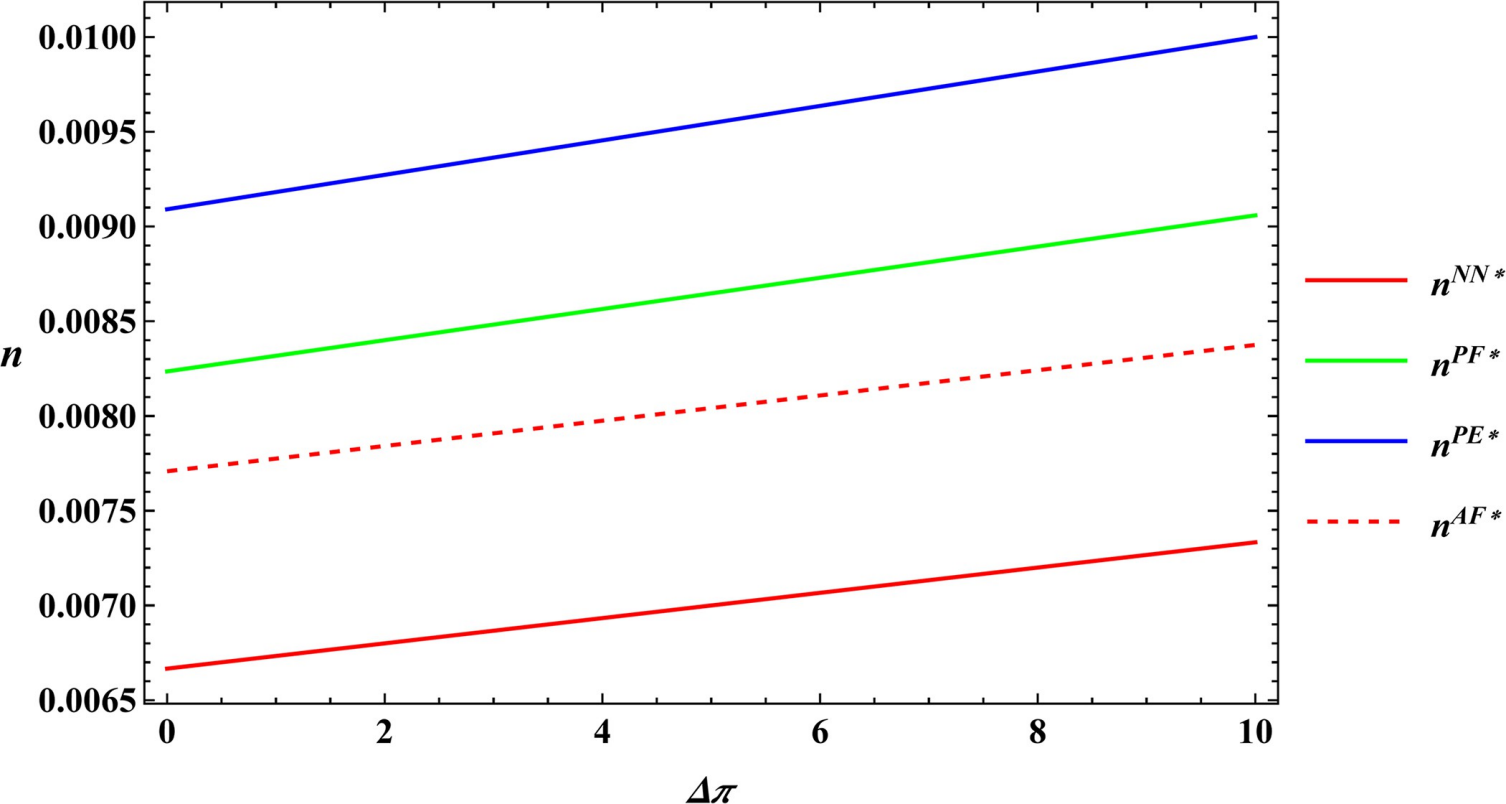
Sensitivity analysis of consumer premiums in farm size.

**Fig 7:**
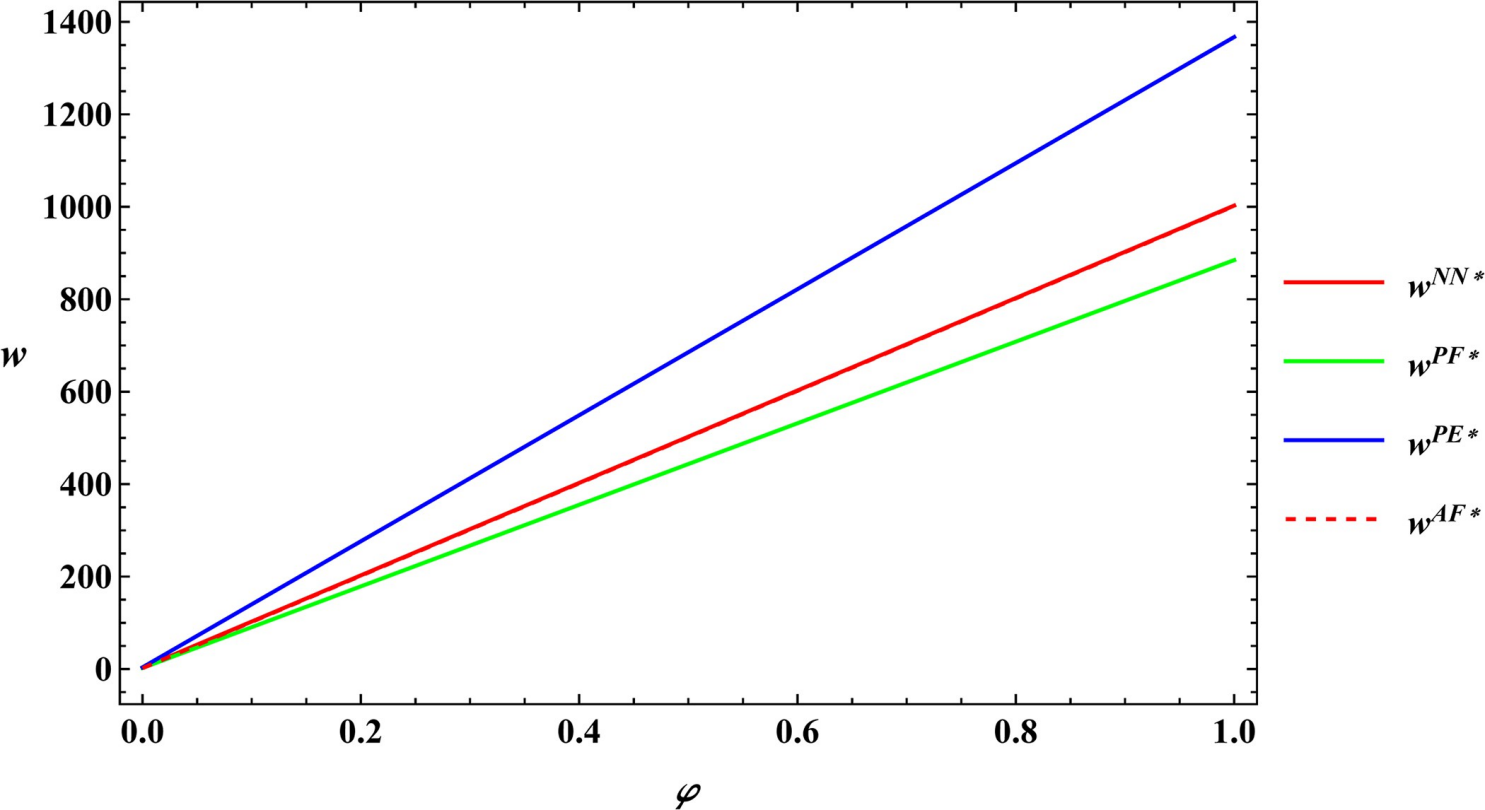
Sensitivity analysis of consumer preference for supporting farmers in the purchase price of agricultural products.

**Fig 8:**
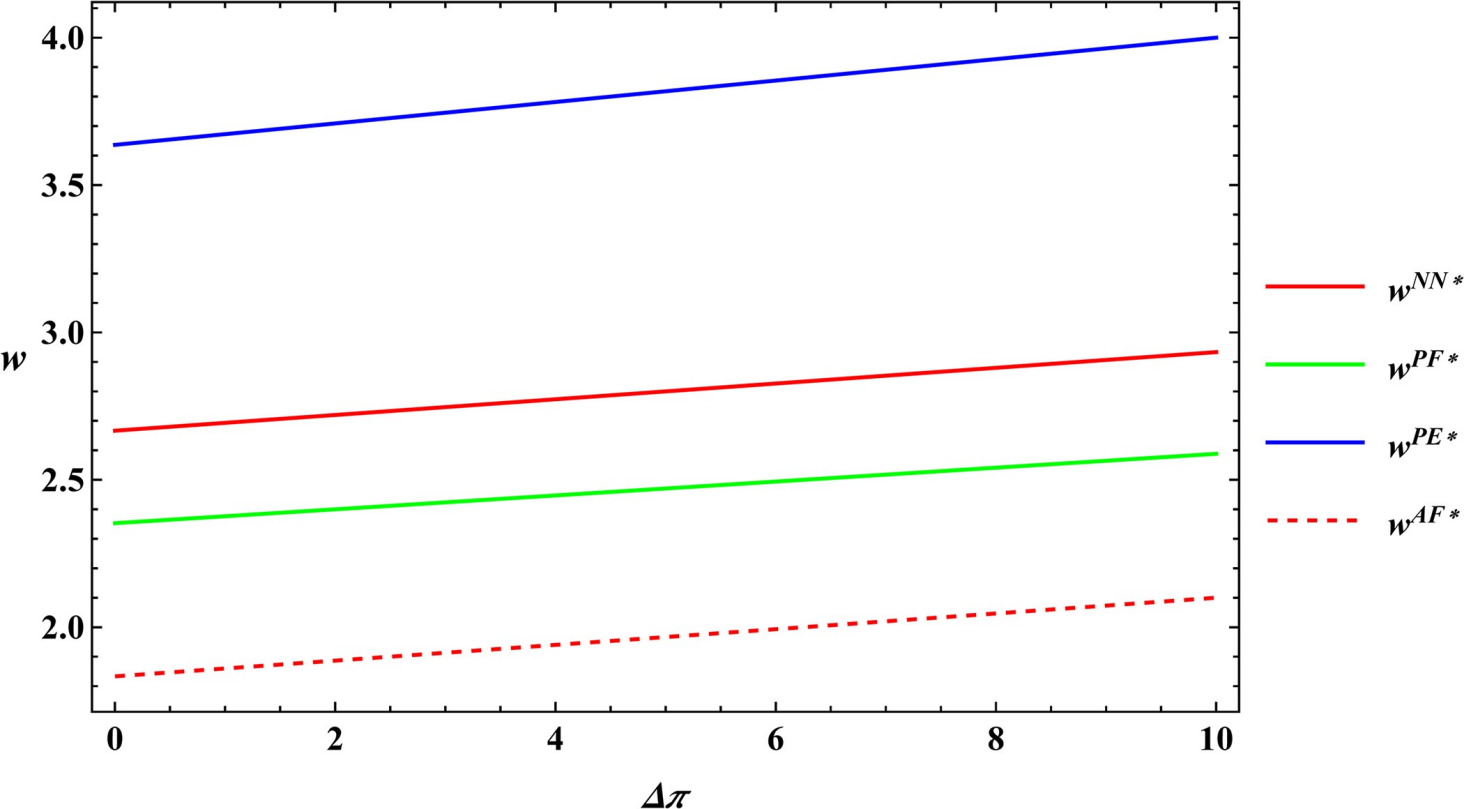
Sensitivity analysis of consumer premiums in the purchase price of agricultural products.

The following numerical simulations further analyze the impact of consumer preference for supporting farmers and consumer premiums on farm size and the purchase price of agricultural products. The parameter values considered are similar to those of Proposition 1, and these values are as follows: α = 8, μ = 5, c_1_ = 10, σ = 1, β = 0.4, θ = 25, y = 20, φ = 0.08, Δπ = 3000. The numerical simulation results for Proposition 2 are shown in Figs 5–8.

According to Proposition 2 and numerical simulations it can be seen that a positive correlation between farm size, the purchase price of agricultural products, and consumer preference for supporting farmers, regardless of the type of subsidies. The situation remains unchanged when it comes to consumer premiums. The conclusion of Proposition 2 is similar to Sun’s study [46], which examined consumer preference for supporting farmers and consumer premiums under consignment and resale models, also showing a positive impact on farm size and the purchase price of agricultural products. Regardless of the subsidy model, there is a positive correlation between farm size, the purchase price of agricultural products, and consumer preference for supporting farmers, as well as consumer premiums. This correlation exists because consumers, driven by their preference for supporting farmers, are willing to pay a premium to help farmers in impoverished areas. As a result, consumers purchase agricultural products through e-commerce platforms at a premium price. Upon receiving these premiums, e-commerce platforms raise the purchase price of agricultural products to share the additional revenue with the farmers. As the purchase price increases, farmers are motivated to invest more in production to achieve higher profits, which in turn leads to an expansion of farm size.

From the above propositions, the following managerial implications can be derived: Consumer preference for supporting farmers benefits both farmers and e-commerce platforms, hence using new media and other means to promote supporting agriculture products so that consumers understand and recognize supporting agriculture products and stimulate consumer preference for supporting agriculture products to ultimately achieve the purpose of helping farmers.

**Proposition 3:**

Proposition 3 discusses the relationship between optimal total production and consumer preference for supporting farmers, consumer premium. The expression for the optimal total production is Q* = n*y.


$$\frac{\partial {Q^{NN}}^{*}}{\partial \varphi }=\frac{\Delta \pi }{2(\mu +\mu \sigma^{4}+2c_{1})}>0,\frac{\partial {Q^{PF}}^{*}}{\partial \varphi }=\frac{(1+\beta )\Delta \pi }{2(1+\beta )\mu (1+\sigma^{4})+4c_{1}}>0$$

$$\frac{\partial {Q^{PE}}^{*}}{\partial \varphi }=\frac{\Delta \pi }{2(\mu +\mu \sigma^{4}+2c_{1}-2\beta c_{1})}>0,\frac{\partial {Q^{AF}}^{*}}{\partial \varphi }=\frac{\Delta \pi }{2(\mu +\mu \sigma^{4}+2c_{1})}>0$$

$$\frac{\partial {Q^{NN}}^{*}}{\partial \Delta \pi }=\frac{\varphi }{2(\mu +\mu \sigma^{4}+2c_{1})}>0,\frac{\partial {Q^{PF}}^{*}}{\partial \Delta \pi }=\frac{(1+\beta )\varphi }{2(1+\beta )\mu (1+\sigma^{4})+4c_{1}}>0$$

$$\frac{\partial {Q^{PE}}^{*}}{\partial \Delta \pi }=\frac{\varphi }{2(\mu +\mu \sigma^{4}+2c_{1}-2\beta c_{1})}>0,\frac{\partial {Q^{AF}}^{*}}{\partial \Delta \pi }=\frac{\varphi }{2(\mu +\mu \sigma^{4}+2c_{1})}>0$$

**Fig 9:**
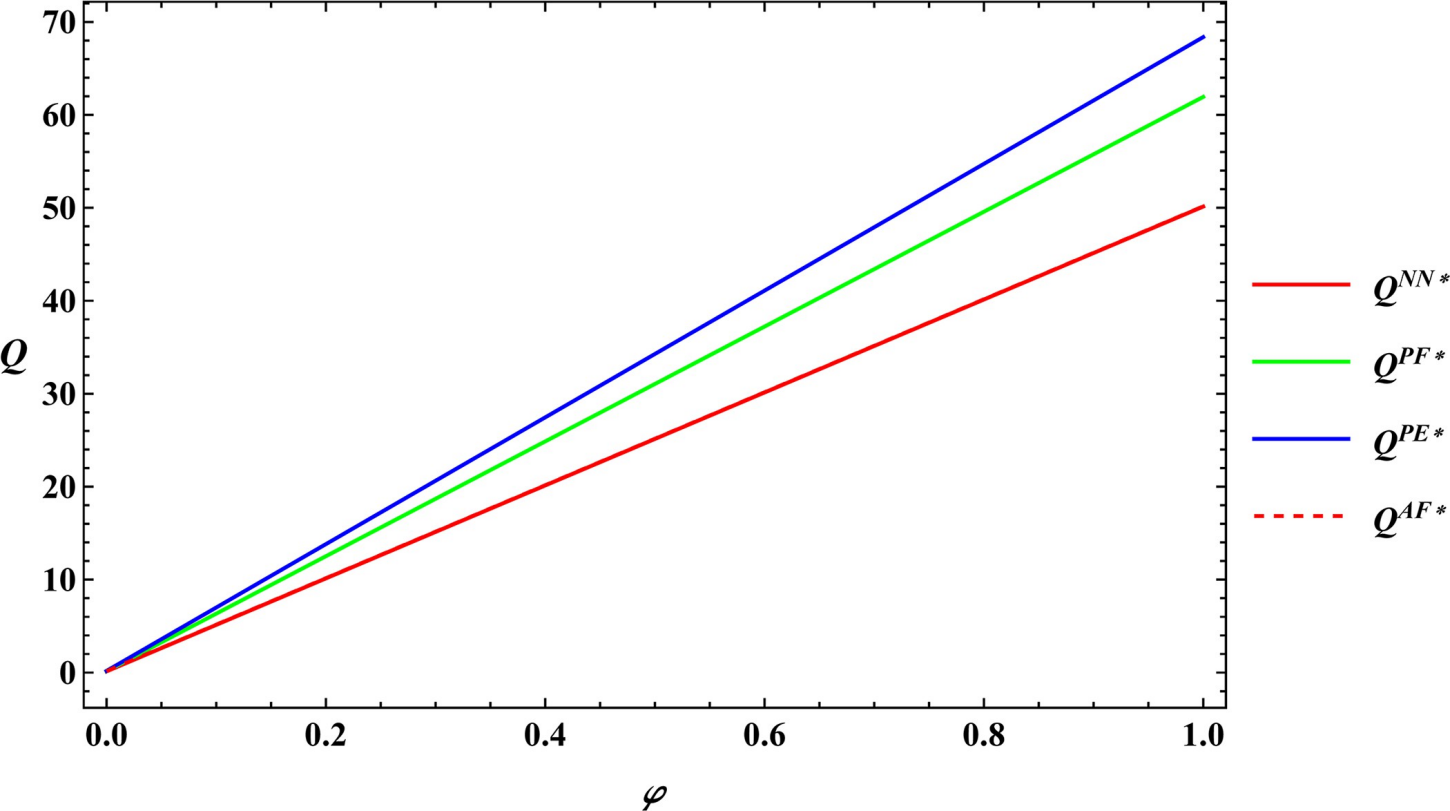
Sensitivity analysis of consumer preference for supporting farmers in optimal total production.

**Fig 10:**
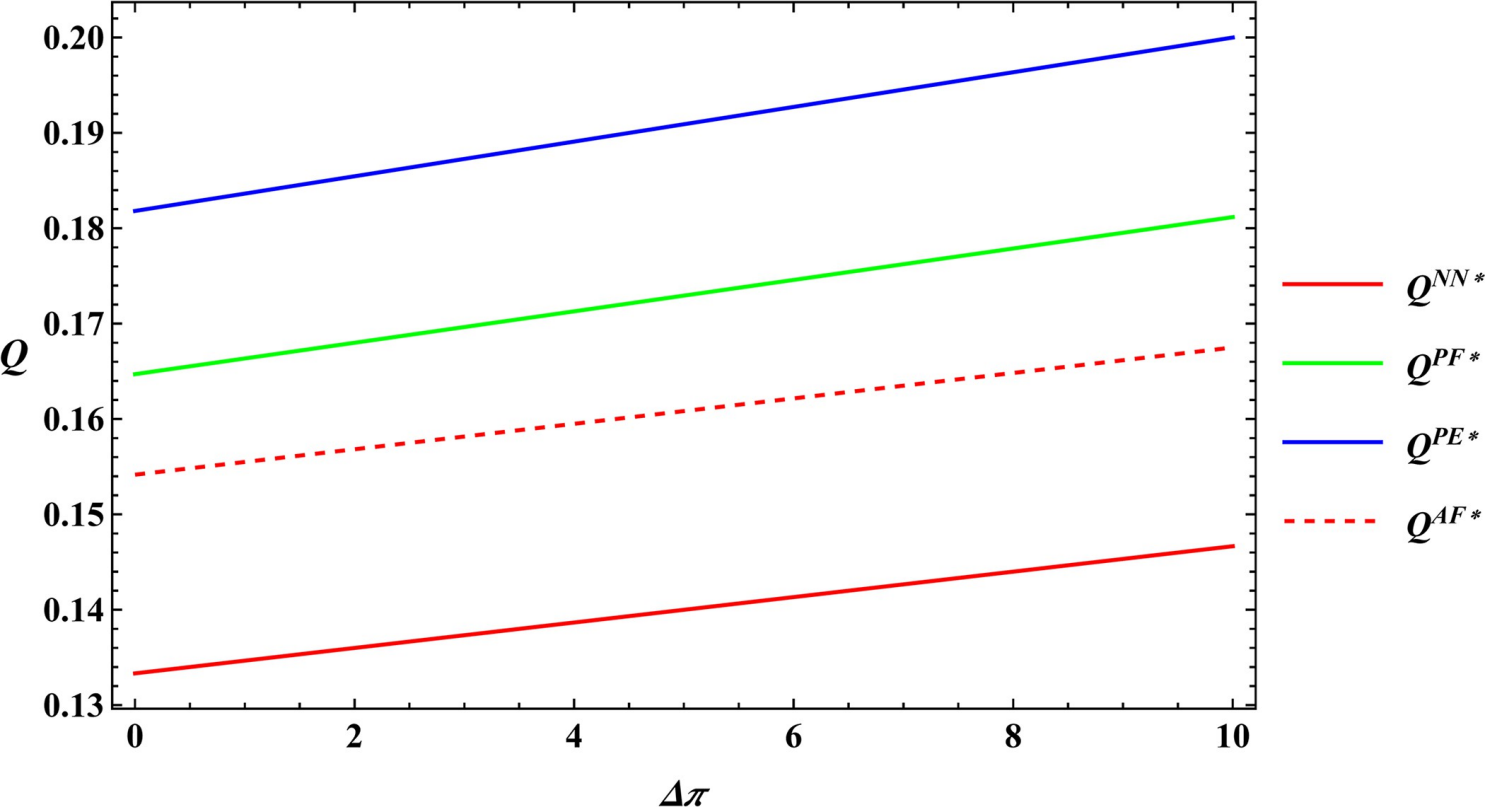
Sensitivity analysis of consumer premiums in optimal total production.

The following numerical simulation will further analyze Proposition 3, using the same parameter values as mentioned earlier. The results are shown in Figs 9 and 10.

Analysis of Proposition 3 and Figs 9 and 10 illustrates that consumer preference for supporting farmers and consumer payment premiums are positively related to total agricultural productions under different government subsidies. The reasons are as follows:

Consumers are willing to pay a premium for these products through e-commerce platform out of their preference to support farmers. The e-commerce platform shares part of the premium with the farmer by increasing the purchase price of agricultural products, thus increasing the farmer’s profits. An increase in farmer profits will encourage the farmer to expand farm size, thereby increasing the total productions. In sum, these observations support Proposition 3.

**Proposition 4:**

$$\frac{\partial {{\pi_{F}}^{NN}}^{*}}{\partial \varphi }=\frac{\Delta \pi (\alpha +\Delta \pi \varphi )c_{1}}{2{\left(\mu +\mu \sigma^{4}+2c_{1}\right)}^{2}}>0,\frac{\partial {{\pi_{F}}^{PF}}^{*}}{\partial \varphi }=\frac{{\left(1+\beta \right)}^{2}\Delta \pi (\alpha +\Delta \pi \varphi )c_{1}}{2{\left((1+\beta )\mu (1+\sigma^{4})+2c_{1}\right)}^{2}}>0$$

$$\frac{\partial {{\pi_{F}}^{PE}}^{*}}{\partial \varphi }=\frac{\Delta \pi (\alpha +\Delta \pi \varphi )c_{1}}{2{\left(\mu (1+\sigma^{4})-2(-1+\beta )c_{1}\right)}^{2}}>0,\frac{\partial {{\pi_{F}}^{AF}}^{*}}{\partial \varphi }=\frac{\Delta \pi (\theta +y(\alpha +\Delta \pi \varphi ))c_{1}}{2y{\left(\mu +\mu \sigma^{4}+2c_{1}\right)}^{2}}>0$$

$$\frac{\partial {{\pi_{F}}^{NN}}^{*}}{\partial \Delta \pi }=\frac{\varphi (\alpha +\Delta \pi \varphi )c_{1}}{2{\left(\mu +\mu \sigma^{4}+2c_{1}\right)}^{2}}>0,\frac{\partial {{\pi_{F}}^{PF}}^{*}}{\partial \Delta \pi }=\frac{{\left(1+\beta \right)}^{2}\varphi (\alpha +\Delta \pi \varphi )c_{1}}{2{\left((1+\beta )\mu (1+\sigma^{4})+2c_{1}\right)}^{2}}>0$$

$$\frac{\partial {{\pi_{F}}^{PE}}^{*}}{\partial \Delta \pi }=\frac{\varphi (\alpha +\Delta \pi \varphi )c_{1}}{2{\left(\mu (1+\sigma^{4})-2(-1+\beta )c_{1}\right)}^{2}}>0,\frac{\partial {{\pi_{F}}^{AF}}^{*}}{\partial \Delta \pi }=\frac{\varphi (\theta +y(\alpha +\Delta \pi \varphi ))c_{1}}{2y{\left(\mu +\mu \sigma^{4}+2c_{1}\right)}^{2}}>0$$

$$\frac{\partial {{\pi_{C}}^{NN}}^{*}}{\partial \varphi }=\frac{\Delta \pi (\alpha +\Delta \pi \varphi )}{2(\mu +\mu \sigma^{4}+2c_{1})}>0,\frac{\partial {{\pi_{C}}^{PF}}^{*}}{\partial \varphi }=\frac{2(1+\beta )\Delta \pi (\alpha +\Delta \pi \varphi )}{4(1+\beta )\mu (1+\sigma^{4})+8c_{1}}>0$$

$$\frac{\partial {{\pi_{C}}^{PE}}^{*}}{\partial \varphi }=\frac{2\Delta \pi (\alpha +\Delta \pi \varphi )}{4\mu (1+\sigma^{4})-8(-1+\beta )c_{1}}>0,\frac{\partial {{\pi_{C}}^{AF}}^{*}}{\partial \varphi }=\frac{\Delta \pi (\theta +y(\alpha +\Delta \pi \varphi ))}{2y(\mu +\mu \sigma^{4}+2c_{1})}>0$$

$$\frac{\partial {{\pi_{C}}^{NN}}^{*}}{\partial \Delta \pi }=\frac{\varphi (\alpha +\Delta \pi \varphi )}{2(\mu +\mu \sigma^{4}+2c_{1})}>0,\frac{\partial {{\pi_{C}}^{PF}}^{*}}{\partial \Delta \pi }=\frac{2(1+\beta )\varphi (\alpha +\Delta \pi \varphi )}{4(1+\beta )\mu (1+\sigma^{4})+8c_{1}}>0$$

$$\frac{\partial {{\pi_{C}}^{PE}}^{*}}{\partial \Delta \pi }=\frac{2\varphi (\alpha +\Delta \pi \varphi )}{4\mu (1+\sigma^{4})-8(-1+\beta )c_{1}}>0,\frac{\partial {{\pi_{C}}^{AF}}^{*}}{\partial \Delta \pi }=\frac{\varphi (\theta +y(\alpha +\Delta \pi \varphi ))}{2y(\mu +\mu \sigma^{4}+2c_{1})}>0$$

**Fig 11:**
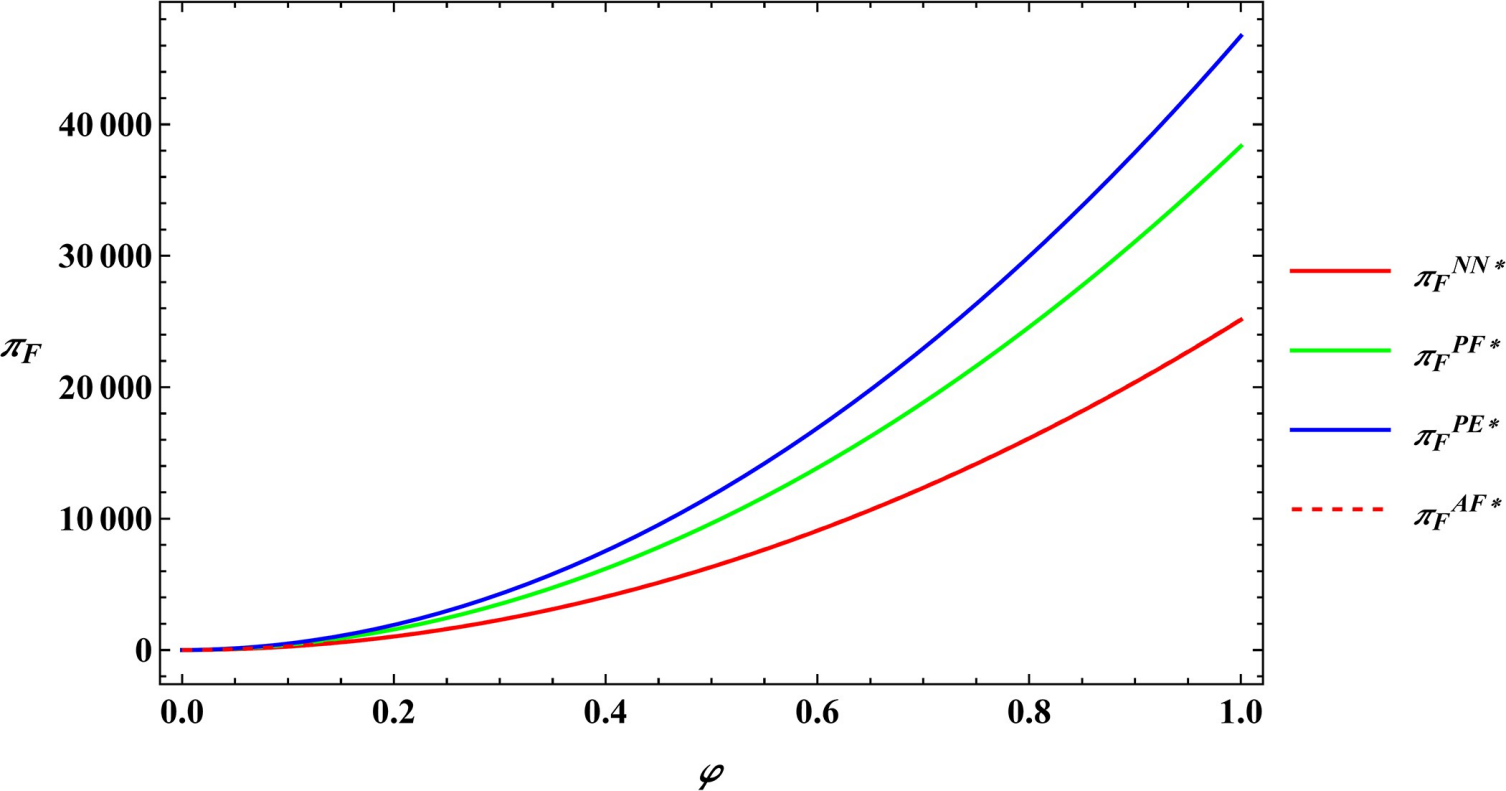
Sensitivity analysis of consumer preference for supporting farmers in the farmer’s optimal profits.

**Fig 12:**
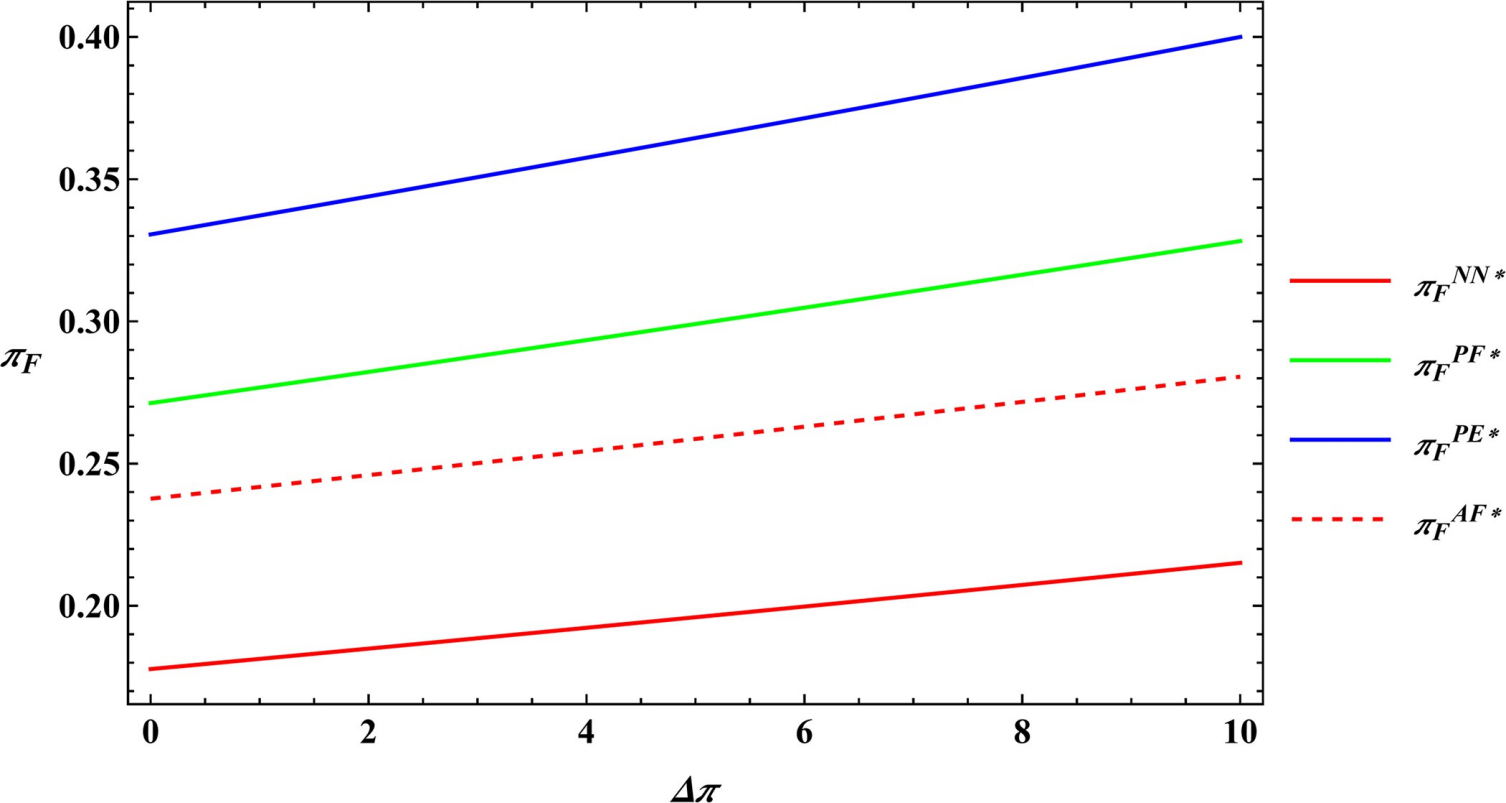
Sensitivity analysis of consumer premiums in the farmer’s optimal profits.

**Fig 13:**
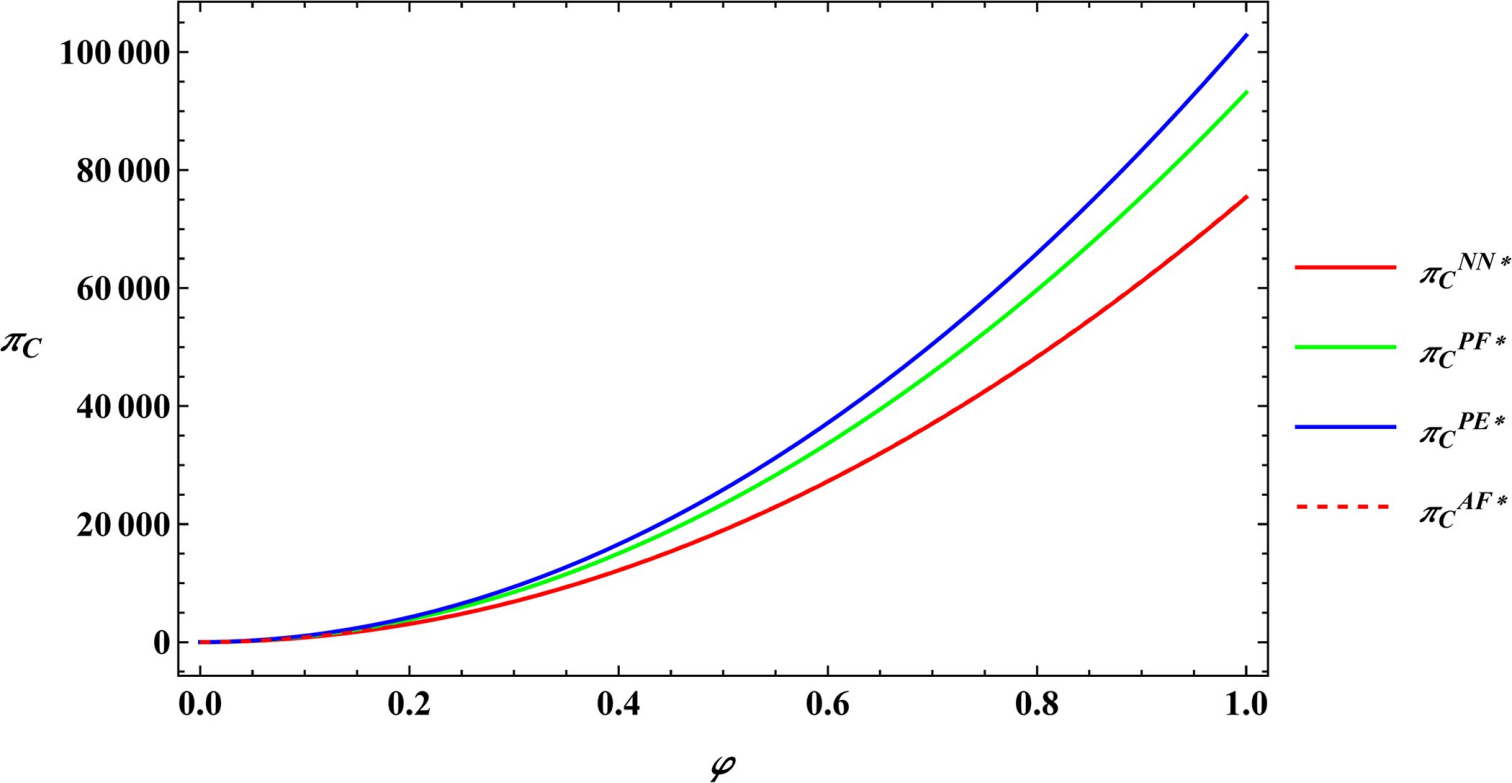
Sensitivity analysis of consumer preference for supporting farmers in the e-commerce platform’s optimal profits.

**Fig 14:**
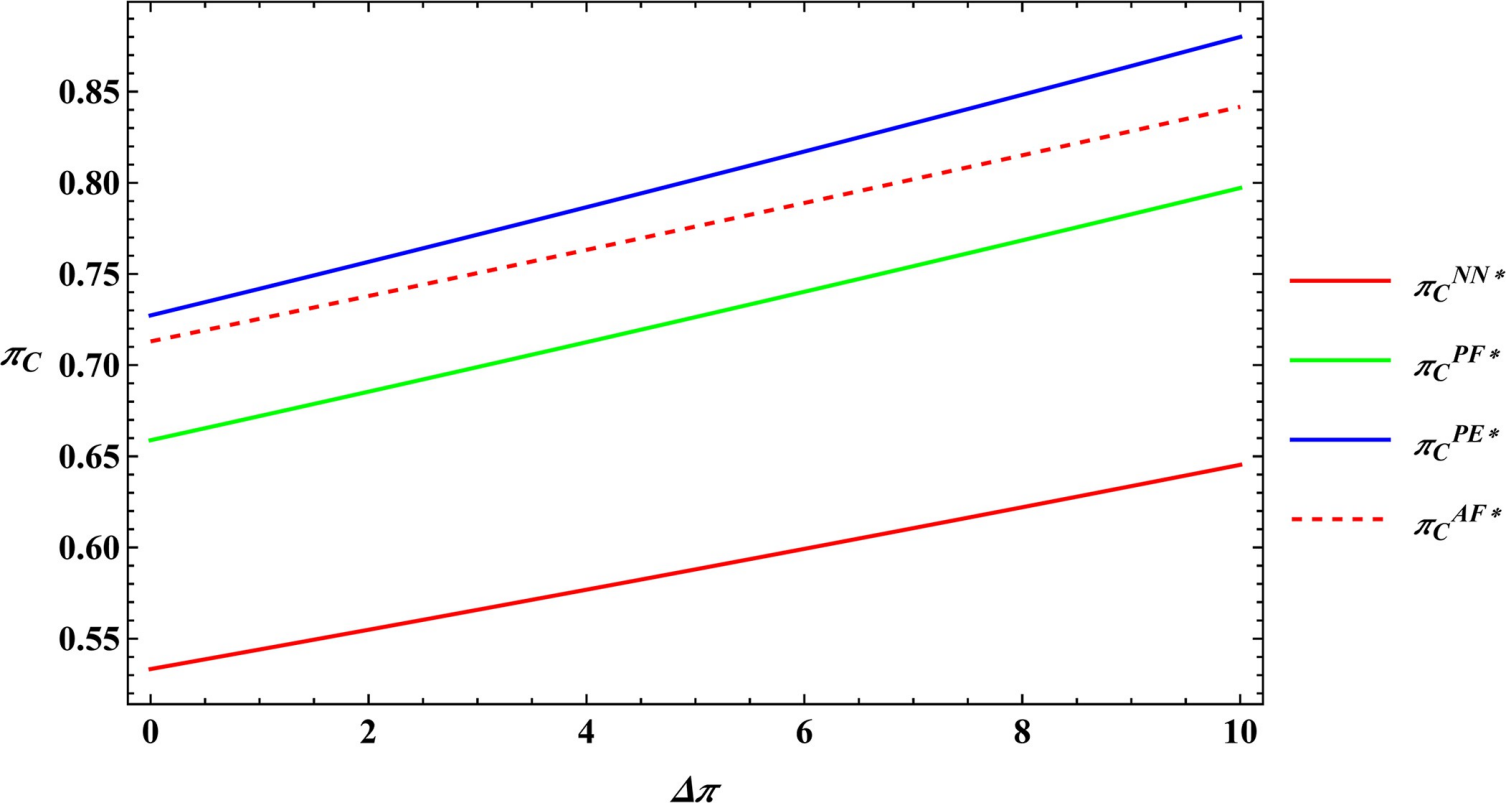
Sensitivity analysis of consumer premiums in the e-commerce platform’s optimal profits.

The parameter values are consistent with those in Proposition 2, and subsequently, numerical simulations are conducted for Proposition 4. The numerical simulation results of Proposition 4 are shown in Figs 11–14.

Proposition 4 and Fig 11–14 demonstrate that, regardless of the type of government subsidies used, consumer preference for supporting farmers is positively related to the optimal profits of both farmers and e-commerce platforms. The same is true for consumer premiums, which are also positively correlated with the optimal profits of farmers and e-commerce platforms. Sun et al. [46] investigated the relationship between consumer preferences for supporting farmers and consumer premiums with the profits of farmers and e-commerce platforms under reselling and agency selling models, finding that in both models, consumer preferences and premiums have a positive correlation with the profits of both farmers and e-commerce platforms. The difference between Proposition 4 and Sun’s conclusions lies in the fact that Proposition 4 analyzes the relationship between consumer preferences for supporting farmers, consumer premiums, and the profits of farmers and e-commerce platforms under different government subsidy models. The reason for the phenomenon observed in Proposition 4 is: Based on the conclusions of Proposition 2 mentioned earlier, consumer preference for supporting farmers leads consumers to be willing to pay a premium for agricultural products. This willingness motivates farmers to expand production and prompts e-commerce platforms to raise the purchase price of agricultural products. As a result, both the optimal profits of farmers and e-commerce platforms increase. This conclusion has also been validated in practice; for example, Alibaba has provided assistance to farmers in Qingjian County, China [55]. Consumers purchase supportive agricultural products from Qingjian County through the Taobao e-commerce platform, benefiting both the farmers in Qingjian County and Alibaba.

**Proposition 5:**

$$\frac{\partial E[{sw}^{NN}]}{\partial \varphi }=\frac{3\Delta \pi (\alpha +\Delta \pi \varphi )}{4(\mu +\mu \sigma^{4}+2c_{1})}>0,\frac{\partial E[{sw}^{PF}]}{\partial \varphi }=\frac{\left(1+\beta )\Delta \pi (\alpha +\Delta \pi \varphi )(3(1+\beta )\mu (1+\sigma^{4})-2(-3+\beta )c_{1}\right)}{4{\left((1+\beta )\mu (1+\sigma^{4})+2c_{1}\right)}^{2}}>0$$

$$\frac{\partial E[{sw}^{PE}]}{\partial \varphi }=\frac{\Delta \pi (\alpha +\Delta \pi \varphi )(3\mu (1+\sigma^{4})+(6-8\beta )c_{1})}{4{\left(\mu (1+\sigma^{4})-2(-1+\beta )c_{1}\right)}^{2}}>0,\frac{\partial E[{sw}^{AF}]}{\partial \varphi }=\frac{2y\Delta \pi \theta +6y^{2}\Delta \pi (\alpha +\Delta \pi \varphi )}{8y^{2}(\mu +\mu \sigma^{4}+2c_{1})}>0$$

$$\frac{\partial E[{sw}^{NN}]}{\partial \Delta \pi }=\frac{3\varphi (\alpha +\Delta \pi \varphi )}{4(\mu +\mu \sigma^{4}+2c_{1})}>0,\frac{\partial E[{sw}^{PF}]}{\partial \Delta \pi }=\frac{\left(1+\beta )\varphi (\alpha +\Delta \pi \varphi )(3(1+\beta )\mu (1+\sigma^{4})-2(-3+\beta )c_{1}\right)}{4{\left((1+\beta )\mu (1+\sigma^{4})+2c_{1}\right)}^{2}}>0$$

$$\frac{\partial E[{sw}^{PE}]}{\partial \Delta \pi }=\frac{\varphi (\alpha +\Delta \pi \varphi )(3\mu (1+\sigma^{4})+(6-8\beta )c_{1})}{4{\left(\mu (1+\sigma^{4})-2(-1+\beta )c_{1}\right)}^{2}}>0,\frac{\partial E[{sw}^{AF}]}{\partial \Delta \pi }=\frac{2y\theta \varphi +6y^{2}\varphi (\alpha +\Delta \pi \varphi )}{8y^{2}(\mu +\mu \sigma^{4}+2c_{1})}>0$$

**Fig 15:**
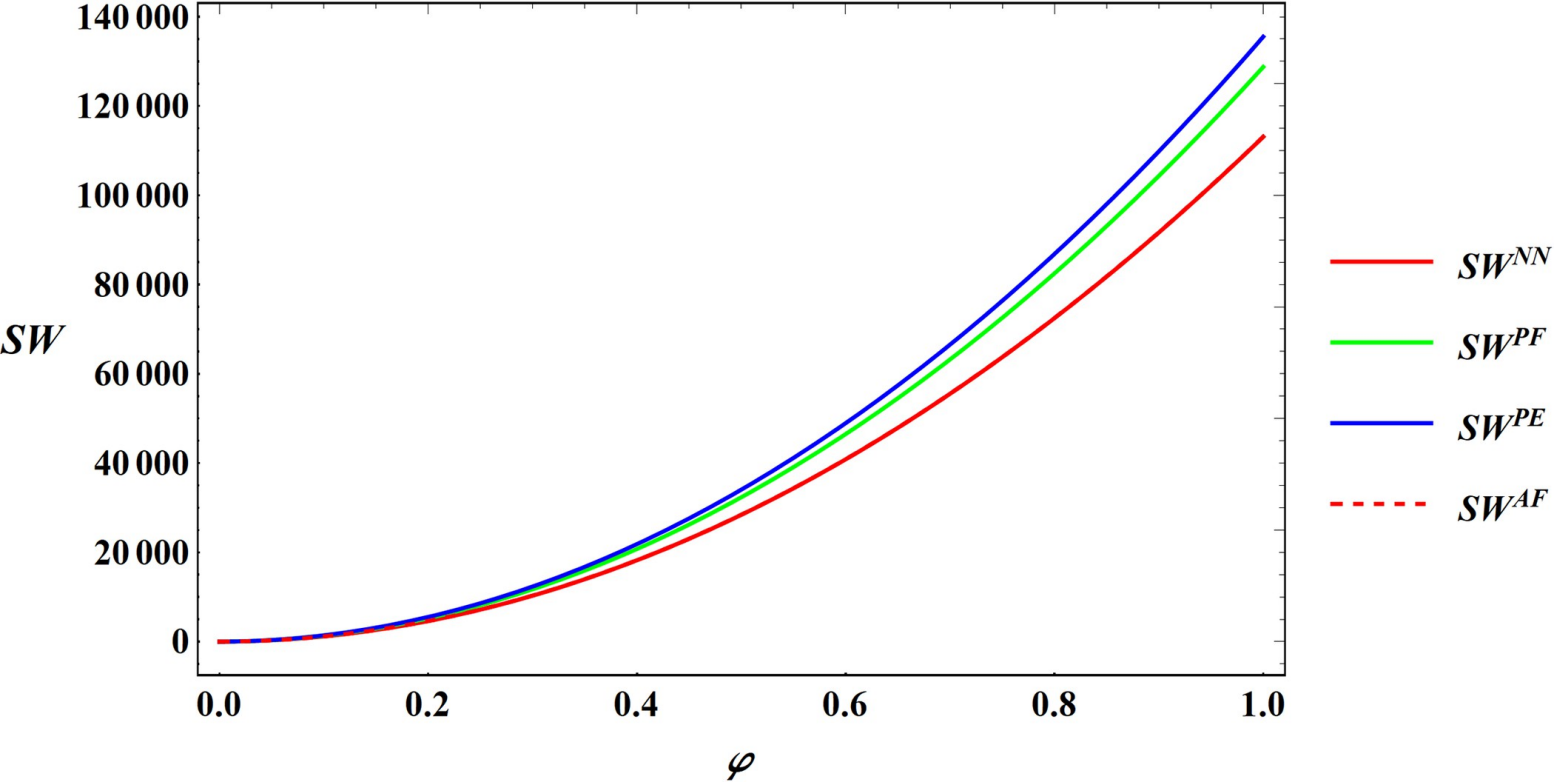
Sensitivity analysis consumer preference for supporting farmers in social welfare.

**Fig 16:**
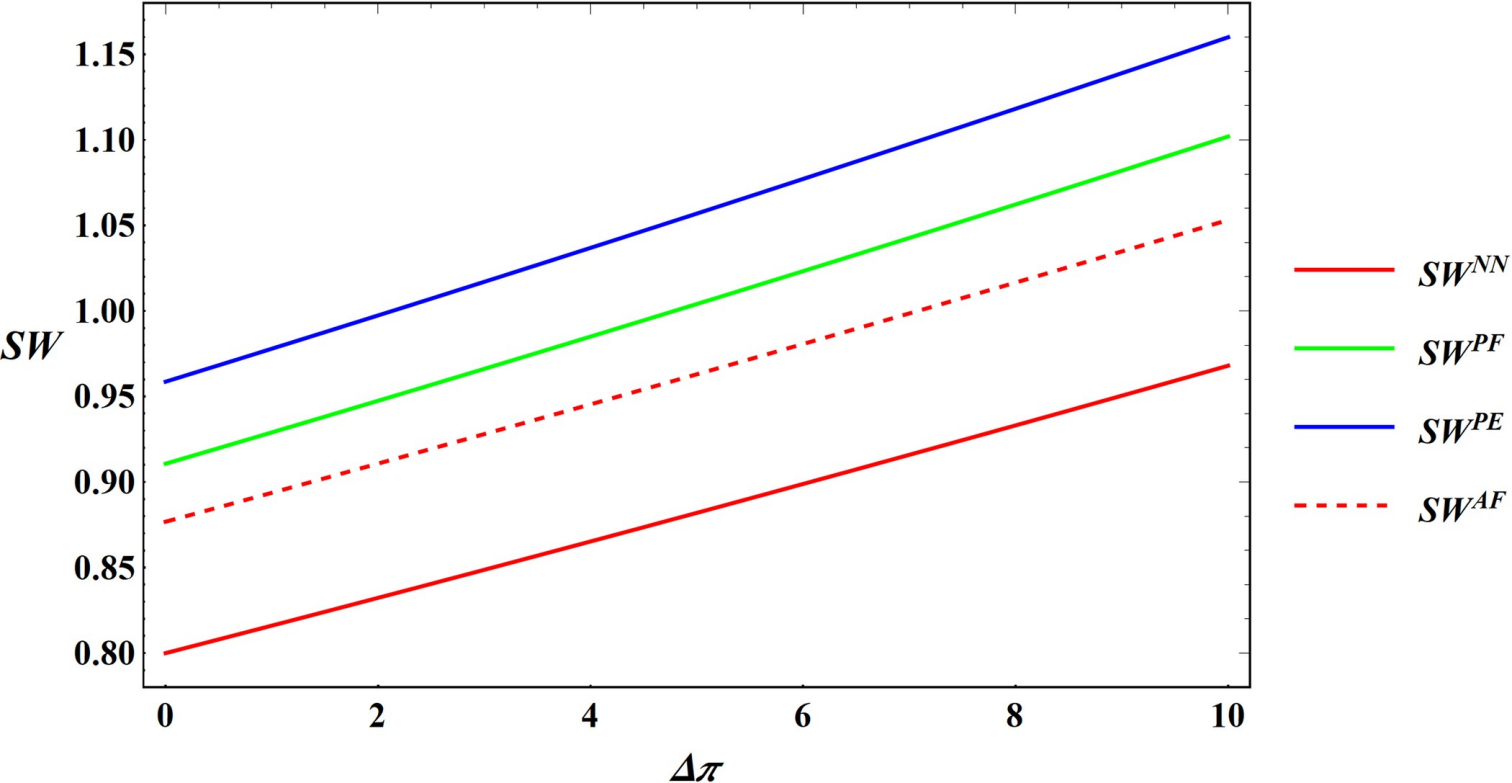
Sensitivity analysis consumer premiums in social welfare.

Numerical simulations of Proposition 5 are conducted with the same parameter settings as in Proposition 2. The results can be found in Figs 15 and 16.

Proposition 5 and Figs 15 amd 16 emphasize that consumer preference for supporting farmers and consumer premiums are consistently positively correlated with expected social welfare. Importantly, this correlation holds regardless of the specific government subsidies approach employed. This relationship can be elucidated as follows:

An increase in consumer preference for supporting farmers and the willingness to pay a premium for agricultural products can act as stimuli for the farmer to expand farm size. Furthermore, since the premium is shared between the e-commerce platform and the farmer, this encourages the e-commerce platform to increase the purchase price of agricultural products. As a result, the profits of the farmer and the e-commerce platform increase. The profits of farmers and e-commerce platforms are important components of social welfare. Therefore, the overall social welfare will escalate as consumer preference for supporting farmers and consumer premiums payments grow.

**Proposition 6:**

$$\frac{\partial E[{sw}^{PF}]}{\partial \beta }>0$$

$$When\;0<\beta \leq \frac{1}{2},\frac{\partial E[{sw}^{PE}]}{\partial \beta }>0$$

$$When\;\frac{1}{2}<\beta <1\;and\;\mu >\frac{-2c_{1}+4\beta c_{1}}{1+\sigma^{4}},\frac{\partial E[{sw}^{PE}]}{\partial \beta }>0$$

$$When\;\frac{1}{2}<\beta <1\;and\;0<\mu <\frac{-2c_{1}+4\beta c_{1}}{1+\sigma^{4}},\frac{\partial E[{sw}^{PE}]}{\partial \beta }<0$$

$$When\;y>\frac{\theta }{\alpha +\Delta \pi \varphi },\frac{\partial E[{sw}^{AF}]}{\partial \theta }>0$$

$$When\;0<y<\frac{\theta }{\alpha +\Delta \pi \varphi },\frac{\partial E[{sw}^{AF}]}{\partial \theta }<0$$

**Proof of Proposition 6:** $\frac{\partial E[{sw}^{PF}]}{\partial \beta }=\frac{{\left(\alpha +\Delta \pi \varphi \right)}^{2}c_{1}((1+\beta )\mu (1+\sigma^{4})-2(-1+\beta )c_{1})}{2{\left((1+\beta )\mu (1+\sigma^{4})+2c_{1}\right)}^{3}}>0,$ E[sw^PE^] and E[sw^AF^] parameters need to be discussed, $\frac{\partial E[{sw}^{PE}]}{\partial \beta }=-\frac{{\left(\alpha +\Delta \pi \varphi \right)}^{2}c_{1}(-\mu (1+\sigma^{4})+(-2+4\beta )c_{1})}{2{\left(\mu (1+\sigma^{4})-2(-1+\beta )c_{1}\right)}^{3}}$, We know from the range of values $2{\left(\mu (1+\sigma^{4})-2(-1+\beta )c_{1}\right)}^{3}>0$, When $0<\beta \leq \frac{1}{2},\;{\left(\alpha +\Delta \pi \varphi \right)}^{2}c_{1}(-\mu (1+\sigma^{4})+(-2+4\beta )c_{1})<0$, so $\frac{\partial E[{sw}^{PE}]}{\partial \beta }>0$. When $\frac{1}{2}<\beta <1$ and $\mu >\frac{-2c_{1}+4\beta c_{1}}{1+\sigma^{4}},\;{\left(\alpha +\Delta \pi \varphi \right)}^{2}c_{1}(-\mu (1+\sigma^{4})+(-2+4\beta )c_{1})<0,\;\frac{\partial E[{sw}^{PE}]}{\partial \beta }>0$. When $\frac{1}{2}<\beta <1$ and $0<\mu <\frac{-2c_{1}+4\beta c_{1}}{1+\sigma^{4}},\;2{\left(\mu (1+\sigma^{4})-2(-1+\beta )c_{1}\right)}^{3}>0$, so $\frac{\partial E[{sw}^{PE}]}{\partial \beta }<0$. The proof process for $\frac{\partial E[{sw}^{AF}]}{\partial \theta }$ is the same.

**Fig 17:**
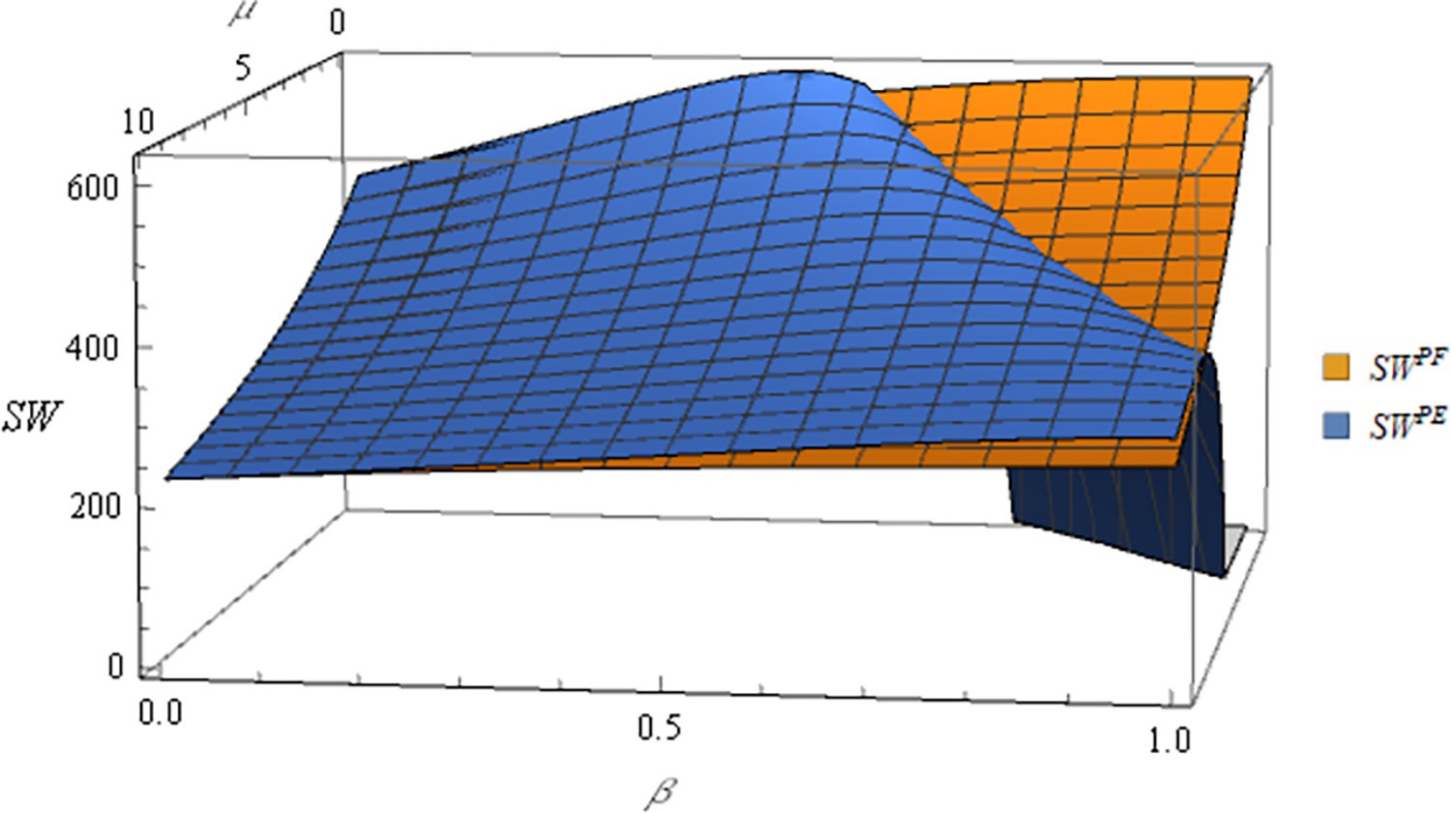
Sensitivity analysis of price elasticity in social welfare under price subsidy.

**Fig 18:**
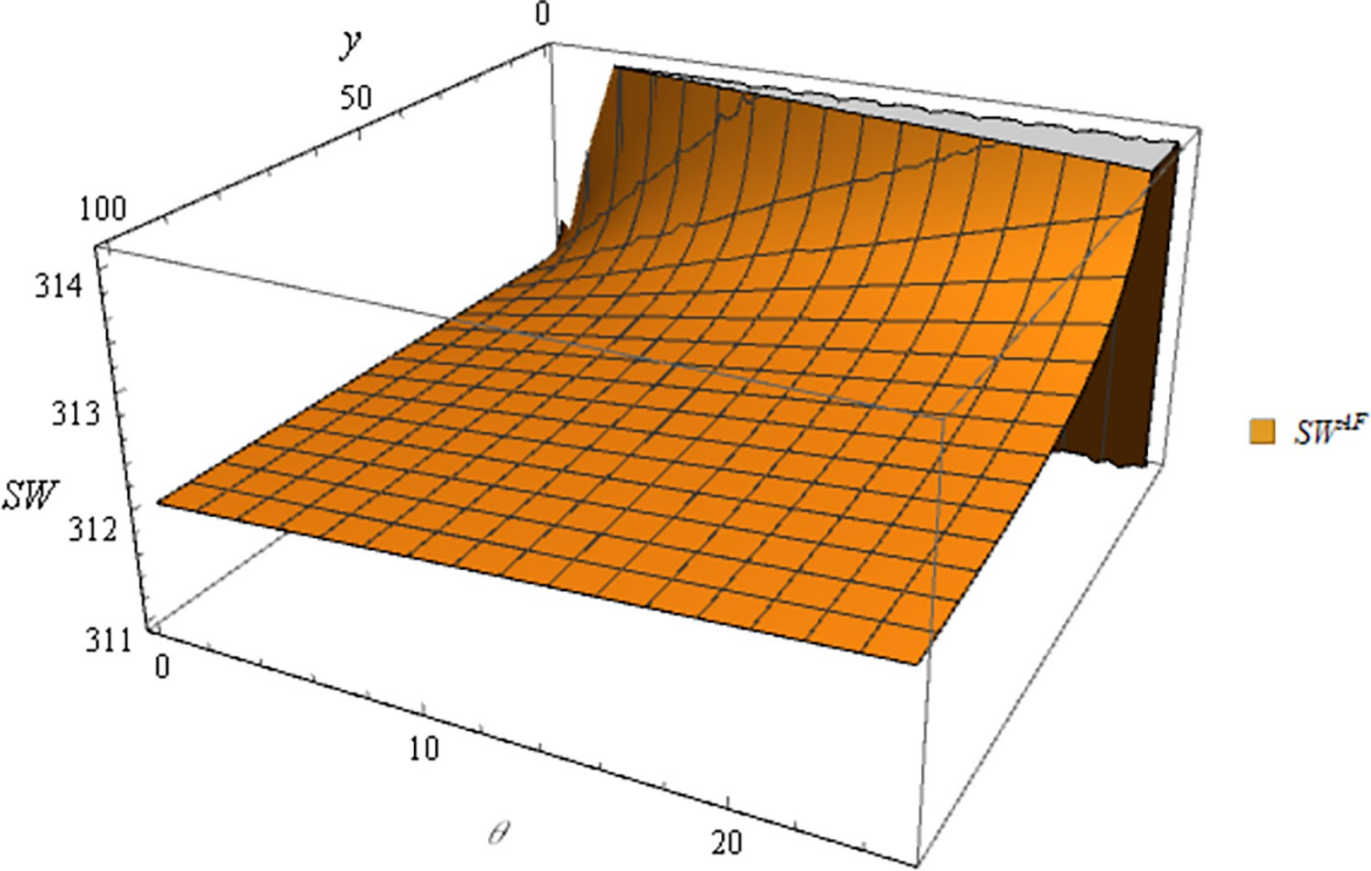
Sensitivity analysis of agricultural productions per unit area in social welfare under area subsidy.

Following this, the numerical simulation for Proposition 6 using the subsequent parameter settings: α = 8, c_1_ = 10, σ = 1, y = 20, φ = 0.05, Δπ = 3000, μ = 5. The numerical simulation results are shown in Figs 17 and 18.

Proposition 6 and Figs 17 and 18 are concluded that different government subsidies methods have varying impacts on social welfare. When the subsidy method is price subsidy and the target is the farmer, government subsidies are positively correlated with expected social welfare. When the subsidies method is price subsidy and the targets are e-commerce platforms, the relationship between government subsidies and expected social welfare depends on the subsidy ratio (β) and the price elasticity coefficient (μ):

When the subsidies ratio is relatively low ($0<\beta \leq \frac{1}{2}$), government subsidies and expected social welfare are positively correlated.

When the subsidies ratio is relatively high ($\frac{1}{2}<\beta <1$), the impact is influenced by the price sensitivity coefficient:

When the price elasticity coefficient is high ($\mu >\frac{-2c_{1}+4\beta c_{1}}{1+\sigma^{4}}$), government subsidies and expected social welfare remain positively correlated.

When the price elasticity coefficient is low ($0<\mu <\frac{-2c_{1}+4\beta c_{1}}{1+\sigma^{4}}$), government subsidies and expected social welfare are negatively correlated.

In situations where subsidies take the form of area subsidy, the relationship between government subsidies and expected social welfare is tied to the agricultural productions per unit area (y):

When the agricultural productions per unit area is high ($y>\frac{\theta }{\alpha +\Delta \pi \varphi }$), government subsidies and expected social welfare are positively correlated.

When the agricultural productions per unit area is low ($0<y<\frac{\theta }{\alpha +\Delta \pi \varphi }$), expected social welfare decreases with increasing government subsidies.

In most cases, government subsidies can facilitate increased income for farmers and e-commerce platforms, thereby leading to a positive correlation between government subsidies and societal welfare in the majority of situations. In certain cases, there can be a negative correlation, which is explained in the case of a negative correlation.

In the price subsidy model with the e-commerce platform as the subsidies target, when the price elasticity coefficient is low, changes in the price of commodities have less impact on demand. In this case, the government subsidizes the e-commerce platform, which raises the purchase price of agricultural products after receiving the subsidies. A portion of these subsidies flows to farmers, incentivizing them to expand production. However, since the price of agricultural products is less affected by demand fluctuations at this point, neither farmer nor e-commerce platform will see a significant increase in their income. In addition, large government subsidies for agricultural products may distort the market and decouple prices from supply and demand, thereby causing farmers to be no longer guided by market signals and thus reducing the efficient allocation of resources. In short, social welfare and government subsidies are negatively correlated in this case. Under the area subsidy model, if the agricultural productions per unit area is low, government subsidies based on the area may lead to inefficient resource allocation and excessively heavier farmers, causing land impoverishment and environmental problems. As a result, the profits of supply chain members will likewise not increase significantly. In this subsidy scenario, government subsidies are negatively correlated with social welfare. In Xie’s study [40], the profit function under the price subsidy model is positively correlated with social welfare, while government subsidies under the area subsidy model are negatively correlated with social welfare. This contrasts with the conclusions drawn in this article. The reasons for the discrepancy between Xie’s conclusions and Proposition 6 are as follows: In the price subsidy model, this study fixes the optimal subsidy ratio of the government, whereas in the area subsidy model, it does not consider the influence of agricultural production per unit area, thus resulting in discrepancies between this study and the present article.

The following managerial implications can be derived: when the government opts for price subsidy, it must carefully consider various factors in formulating its subsidy policies. This requires balancing subsidies ratios to ensure that subsidies are distributed appropriately. Furthermore, when the government adopts area subsidy, the government and the e-commerce platform have to support the farmer. Government can encourage e-commerce platform use digital farm management tools, including agricultural technologies such as remote monitoring, sensing technology, and Internet of Things devices to monitor farmland and agricultural productions conditions. This technical support enables farmer to manage the agricultural productions process better, improving yields and product quality. In addition, farmers training programs can be implemented to teach them modern farming techniques and best practices and encourage them to increase their agricultural production per unit area.

### 6.2. Comparative analysis

In the comparative analysis, this paper aims to provide decision-making guidance for the government and supply chain members by comparing farm size, the purchase price of agricultural products, and the profits of supply chain members under different subsidy models. To ensure the coherence of conclusions in the comparative analysis section, numerical simulations were uniformly conducted after the completion of all comparative analysis findings.

**Proposition 7:**

For ease of description, assume that $M_{1}=\frac{\theta (\mu (1+\sigma^{4})-2(-1+\beta )c_{1})}{2\beta (\alpha +\Delta \pi \varphi )c_{1}}\;M_{2}=\frac{\theta ((1+\beta )\mu (1+\sigma^{4})+2c_{1})}{2\beta (\alpha +\Delta \pi \varphi )c_{1}}$

$$When\;0<y<M_{1},{n^{AF}}^{*}>{n^{PE}}^{*}>{n^{PF}}^{*}>{n^{NN}}^{*}$$

$$When\;M_{1}<y<M_{2},{n^{PE}}^{*}>{n^{AF}}^{*}>{n^{PF}}^{*}>{n^{NN}}^{*}$$

$$When\;y>M_{2},{n^{PE}}^{*}>{n^{PF}}^{*}>{n^{AF}}^{*}>{n^{NN}}^{*}$$

**Proof of Proposition 7.** ${n^{PF}}^{*}-{n^{NN}}^{*}=\frac{\beta (\alpha +\Delta \pi \varphi )c_{1}}{y(\mu +\mu \sigma^{4}+2c_{1})((1+\beta )\mu (1+\sigma^{4})+2c_{1})}>0$, so n^PF*^>n^NN*^.Then, ${n^{PE}}^{*}-{n^{NN}}^{*}=\frac{\beta (\alpha +\Delta \pi \varphi )c_{1}}{y(\mu +\mu \sigma^{4}+2c_{1})(\mu (1+\sigma^{4})-2(-1+\beta )c_{1})}>0$, so n^PE*^>n^NN*^. ${n^{PE}}^{*}-{n^{PF}}^{*}=\frac{\beta^{2}(\alpha +\Delta \pi \varphi )c_{1}}{y((1+\beta )\mu (1+\sigma^{4})+2c_{1})(\mu (1+\sigma^{4})-2(-1+\beta )c_{1})}>0$, so n^PE*^>n^PF*^. At this point we know that ${n^{PE}}^{*}>{n^{PF}}^{*}>{n^{NN}}^{*}.\;{n^{AF}}^{*}-{n^{NN}}^{*}=\frac{\theta }{2y^{2}(\mu +\mu \sigma^{4}+2c_{1})}>0$, so ${n^{AF}}^{*}>{n^{NN}}^{*}.\;{n^{AF}}^{*}-{n^{PF}}^{*}=\frac{(1+\beta )\theta \mu (1+\sigma^{4})-2(-\theta +y\beta (\alpha +\Delta \pi \varphi ))c_{1}}{2y^{2}(\mu +\mu \sigma^{4}+2c_{1})((1+\beta )\mu (1+\sigma^{4})+2c_{1})}$, easy to know that denominator of a fraction $2y^{2}(\mu +\mu \sigma^{4}+2c_{1})((1+\beta )\mu (1+\sigma^{4})+2c_{1})>0$, When y>M_2_, $(1+\beta )\theta \mu (1+\sigma^{4})-2(-\theta +y\beta (\alpha +\Delta \pi \varphi ))c_{1})<0,\;{n^{PF}}^{*}>{n^{AF}}^{*}$, When 0<y<M_2_, $(1+\beta )\theta \mu (1+\sigma^{4})-2(-\theta +y\beta (\alpha +\Delta \pi \varphi ))c_{1})>0,\;{n^{PF}}^{*}<{n^{AF}}^{*},\;{n^{AF}}^{*}-{n^{PE}}^{*}=\frac{\theta \mu (1+\sigma^{4})-2((-1+\beta )\theta +y\beta (\alpha +\Delta \pi \varphi ))c_{1}}{2y^{2}(\mu +\mu \sigma^{4}+2c_{1})(\mu (1+\sigma^{4})-2(-1+\beta )c_{1})}$, Equally easy to know the denominator $2y^{2}(\mu +\mu \sigma^{4}+2c_{1})(\mu (1+\sigma^{4})-2(-1+\beta )c_{1})>0$. When y>M_1_, $\theta \mu (1+\sigma^{4})-2(\left(-1+\beta )\theta +y\beta (\alpha +\Delta \pi \varphi \right))c_{1}<0,\;{n^{AF}}^{*}<{n^{PE}}^{*}.$ When 0<y<M_1_, $\theta \mu (1+\sigma^{4})-2(\left(-1+\beta )\theta +y\beta (\alpha +\Delta \pi \varphi \right))c_{1}>0,\;{n^{AF}}^{*}>{n^{PE}}^{*}.$ M_2_>M_1_. End of proof. The proof process for the comparative analysis below is the same as for Proposition 7.

Assuming 0<y<M_2_ represents low agricultural productions per unit area, M_1_<y<M_2_ represents moderate agricultural productions per unit area, and high agricultural productions per unit area are assumed when y>M_2_.

According to Proposition 7, it can be observed that: farm sizes remain minimal regardless of the agricultural productions per unit area in the unsubsidized model. when the no-government-subsidies model is in place, farmers are reluctant to expand production based on risk-averse preferences, resulting in smaller farm sizes than the government-subsidized model.

The farm size under the price subsidy model varies with changes in the agricultural productions per unit area. However, regardless of changes in agricultural productions per unit area, the farm size of the subsidized e-commerce platform is more significant than the subsidized farmer model. This is because government subsidies to e-commerce platforms enable farmers to receive subsidies through the trickle-down effect, mobilizing their production incentives. When agricultural productions per unit area are low, the farm size of price subsidized is between area-subsidized and unsubsidized models. When the agricultural productions per unit area is moderate, the farm size with price subsidy targeting the e-commerce platform is more significant than other subsidies models, the farm size with price subsidy targeting the farmer is between area subsidy and unsubsidized. When the agricultural productions per unit area is high, the farm size in the price subsidized model is larger than the area-subsidized and unsubsidized. Thus, the farm size is not only affected by the government’s subsidies but is also inextricably linked to agricultural productions per unit area.

Farm size under the area subsidy model is the largest when agricultural productions per unit area is low. Farmers will prioritize expanding their acreage to receive more government subsidies. However, at this point, the government needs to be aware that the farmer’s objective is to expand the farm size more rather than focusing on maximizing the agricultural productions per unit area. This will result in a certain amount of land wastage. When the agricultural productions per unit area is moderate, choosing the model of price subsidy to the e-commerce platform will maximize the farm size. When the agricultural productions per unit area is high, the government still selects price subsidy where the subsidy target is the e-commerce platform that maximizes the farm size. When the agricultural productions per unit area is moderate or high, the government only needs to set the appropriate subsidies price to subsidize the e-commerce platform, which will maximize the efficiency of contract farming.

**Proposition 8:**

For ease of description, assume that $M_{3}=\frac{\theta (\mu +\mu \sigma^{4}+c_{1})((1+\beta )\mu (1+\sigma^{4})+2c_{1})}{\beta \mu (1+\sigma^{4})(\alpha +\Delta \pi \varphi )c_{1}}$

$$When\;0<y<M_{3},{w^{PE}}^{*}>{w^{NN}}^{*}>{w^{PF}}^{*}>{w^{AF}}^{*}$$

$$When\;y>M_{3},{w^{PE}}^{*}>{w^{NN}}^{*}>{w^{AF}}^{*}>{w^{PF}}^{*}$$

The assumptions of Proposition 8 are to categorize agricultural productions per unit area into higher and lower ranges.

Proposition 8 shows that when the agricultural productions per unit area is low, the purchase price of agricultural products under price subsidy where the subsidy target is the e-commerce platform is the highest, and the purchase price of agricultural products under other subsidized modes are in the following in: unsubsidized mode, price subsidy where the subsidy target is the farmer mode, and area subsidy mode. However, when the agricultural productions per unit area is higher, the purchase price of agricultural products under the area subsidy mode will be higher than that of agricultural products under the price subsidy farmer mode. The relationship between the purchase price of agricultural products under the other subsidies modes remain the same as when agricultural productions per unit area is low.

The reason for this is that if the government subsidizes the e-commerce platform, the e-commerce platform will share the subsidies with the farmer by increasing the purchase price of agricultural products. However, when the government subsidies are targeted at the farmer, the minimum value of the purchase price of the agricultural products that the farmer can afford to pay will be lower accordingly. Meanwhile, the price subsidy model is usually associated with quality certification and standards. E-commerce platforms may be willing to pay higher prices if the produce meets high quality standards.

**Proposition 9:**

For ease of description, assume that: $M_{4}=\frac{\theta ((1+\beta )\mu (1+\sigma^{4})+2c_{1})}{2\beta (\alpha +\Delta \pi \varphi )c_{1}}\;M_{5}=\frac{\theta (\mu (1+\sigma^{4})-2(-1+\beta )c_{1})}{2\beta (\alpha +\Delta \pi \varphi )c_{1}}$

$$When\;0<y<M_{5},{{\pi_{F}}^{AF}}^{*}>{{\pi_{F}}^{PE}}^{*}>{{\pi_{F}}^{PF}}^{*}>{{\pi_{F}}^{NN}}^{*}$$

$${When\;M}_{5}<y<M_{4},{{\pi_{F}}^{PE}}^{*}>{{\pi_{F}}^{AF}}^{*}>{{\pi_{F}}^{PF}}^{*}>{{\pi_{F}}^{NN}}^{*}$$

$$When\;y>M_{4},{{\pi_{F}}^{PE}}^{*}>{{\pi_{F}}^{PF}}^{*}>{{\pi_{F}}^{AF}}^{*}>{{\pi_{F}}^{NN}}^{*}$$

Drawing on the above assumptions, agricultural productions per unit area were categorized into high, moderate, and low ranges.

According to Proposition 9, it can be concluded that: when the agricultural productions per unit area is low, the highest profit of the area subsidy method of farmers, the lowest profit of unsubsidized farmers, the profit of the price subsidy method between the area subsidy and unsubsidized, the price subsidy method of subsidies for the e-commerce platform is higher than the subsidies object for the farmer’s profit. Reference 40 [40] found that under the area subsidy model, farmers’ profits are consistently higher than under the price subsidy model, which differs from Proposition 9. The reason for this discrepancy is that Reference 40 did not consider the per unit area production factor, this paper incorporates the factor of unit area production. Proposition 9 is due to the following reasons:

Low agricultural productions per unit area may be accompanied by higher production risks when agricultural productions per unit area is low. Price subsidy is usually dependent on market prices, and if market prices are volatile, farmers with low yields may be exposed to a greater risk of price fluctuations. Area subsidy is relatively stable and unaffected by market price fluctuations and therefore may reduce risk and increase steady incomes for farmers. Secondly, area subsidy may encourage farmers to diversify their cultivation and reduce their dependence on a single agricultural product. This helps to reduce the impact of market price volatility on farmers and improves the stability of the overall agricultural system.

When the agricultural productions per unit area is moderate, at this time in accordance with the price subsidy and subsidy target is the e-commerce platform for the highest profits of the farmer, the profits of the farmer with unsubsidized are still the lowest, area subsidy and price subsidy for farmer between the two and the profits of farmers under the area subsidy model is higher than the price subsidy for the farmers under the profits of farmers.

When the agricultural productions per unit area is higher, farmers’ profit under the price subsidy e-commerce platform is higher than that of farmers under the price subsidy model, followed by the gain of farmers under the area subsidy. Finally, farmers’ profit under unsubsidized model is still the lowest. When the agricultural productions per unit area increases, the profit of the farmer under the area subsidy approach decreases because as the yield increases, the farmer may need to invest more resources, such as fertilizers, chemicals, and labor, and as a result, the farm size decreases, so the amount of the area subsidy decreases, and the profit of the farmer decreases. It’s worth noting that the profit of government-subsidized e-commerce platform farmers under the price-subsidies model is consistently higher than that of government-subsidized farmers, regardless of the agricultural productions per unit area. When the government subsidizes an e-commerce platform, the e-commerce platform can promote rural employment and income growth, which can drive the growth of farmers’ profits and improve the economic vitality of the whole region.

**Proposition 10:**

The following assumptions are given:

$$L_{1}=c_{1}(2\theta +\beta (\alpha +\Delta \pi \varphi )\sqrt{\frac{\left(1+\beta )\theta^{2}(\mu +\mu \sigma^{4}+2c_{1})((1+\beta )\mu (1+\sigma^{4})+2c_{1}\right)}{\beta^{2}{\left(\alpha +\Delta \pi \varphi \right)}^{2}c_{1}^{2}}})$$

$$L_{2}=\left(-2(-1+\beta )\theta +\beta (\alpha +\Delta \pi \varphi )\sqrt{\frac{\theta^{2}(\mu +\mu \sigma^{4}+2c_{1})(\mu (1+\sigma^{4})-2(-1+\beta )c_{1})}{\beta^{2}{\left(\alpha +\Delta \pi \varphi \right)}^{2}c_{1}^{2}}}\right)$$

$$M_{6}=\frac{(1+\beta )\theta \mu (1+\sigma^{4})+L_{1}}{2\beta (\alpha +\Delta \pi \varphi )c_{1}}\;M_{7}=\frac{\theta \mu +\theta \mu \sigma^{4}+c_{1}L_{2}}{2\alpha \beta c_{1}+2\beta \Delta \pi \varphi c_{1}}$$

Proposition 10 reads as follows:

$$When\;0<y<M_{7},{{\pi_{C}}^{AF}}^{*}>{{\pi_{C}}^{PE}}^{*}>{{\pi_{C}}^{PF}}^{*}>{{\pi_{C}}^{NN}}^{*}$$

$$When\;M_{7}<y<M_{6},{{\pi_{C}}^{PE}}^{*}>{{\pi_{C}}^{AF}}^{*}>{{\pi_{C}}^{PF}}^{*}>{{\pi_{C}}^{NN}}^{*}$$

$$When\;y>M_{6},{{\pi_{C}}^{PE}}^{*}>{{\pi_{C}}^{PF}}^{*}>{{\pi_{C}}^{AF}}^{*}>{{\pi_{C}}^{NN}}^{*}$$

Again, similar to the previous assumptions, agricultural productions per unit area are categorized into high, medium, and low ranges.

Proposition 10 is similar to proposition 9 because both farmer and e-commerce platform have similar profit structures, and the relationship between farmers and e-commerce platforms is similar to a cooperative agreement under the contract farming model, which results in identical profit structures for both supply chain subjects. Secondly, the fiercely competitive market environment compels e-commerce platforms to adopt market operation methods similar to those of farmers. This enables them to attract more farmers’ participation and maintain market competitiveness. In summary, this leads to the comparison of the profit function of the e-commerce platform and the comparison of the farmers’ profit under different subsidies modes is the same.

### 6.3 Numerical simulation for comparative analysis

**Fig 19:**
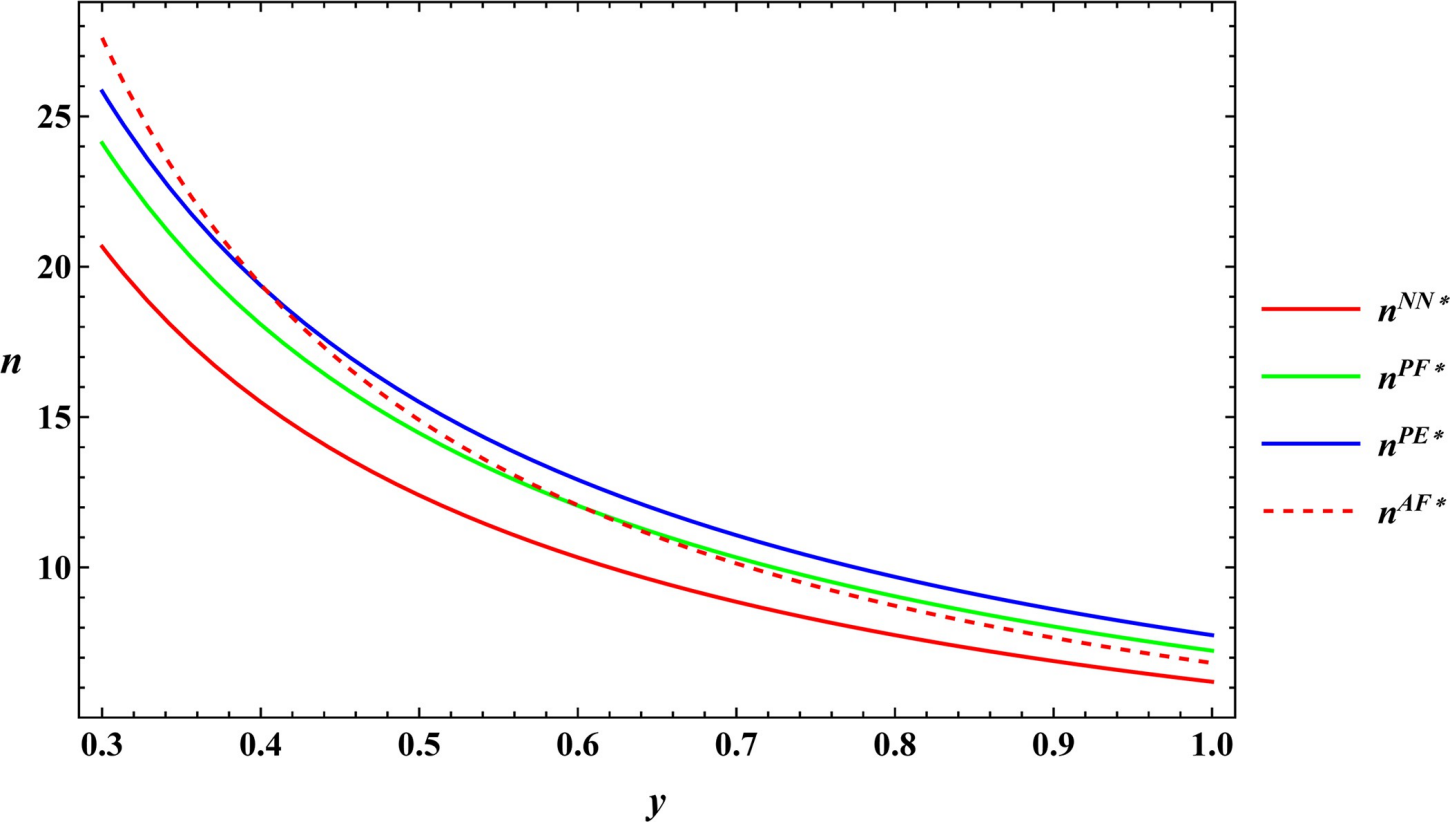
Comparative analysis of farm size.

**Fig 20:**
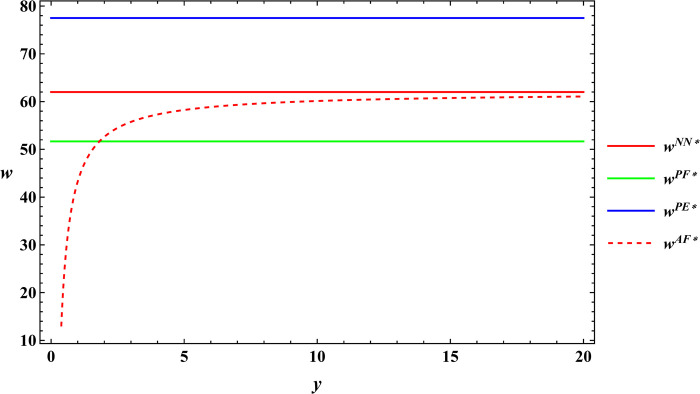
Comparative analysis of the purchase price of agricultural products.

**Fig 21:**
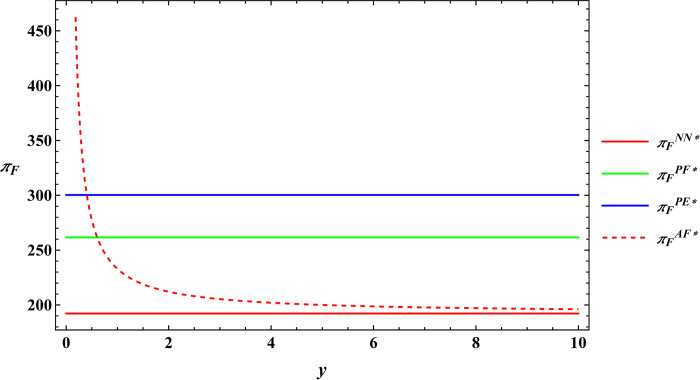
Comparative analysis of optimal profits for the farmer.

**Fig 22:**
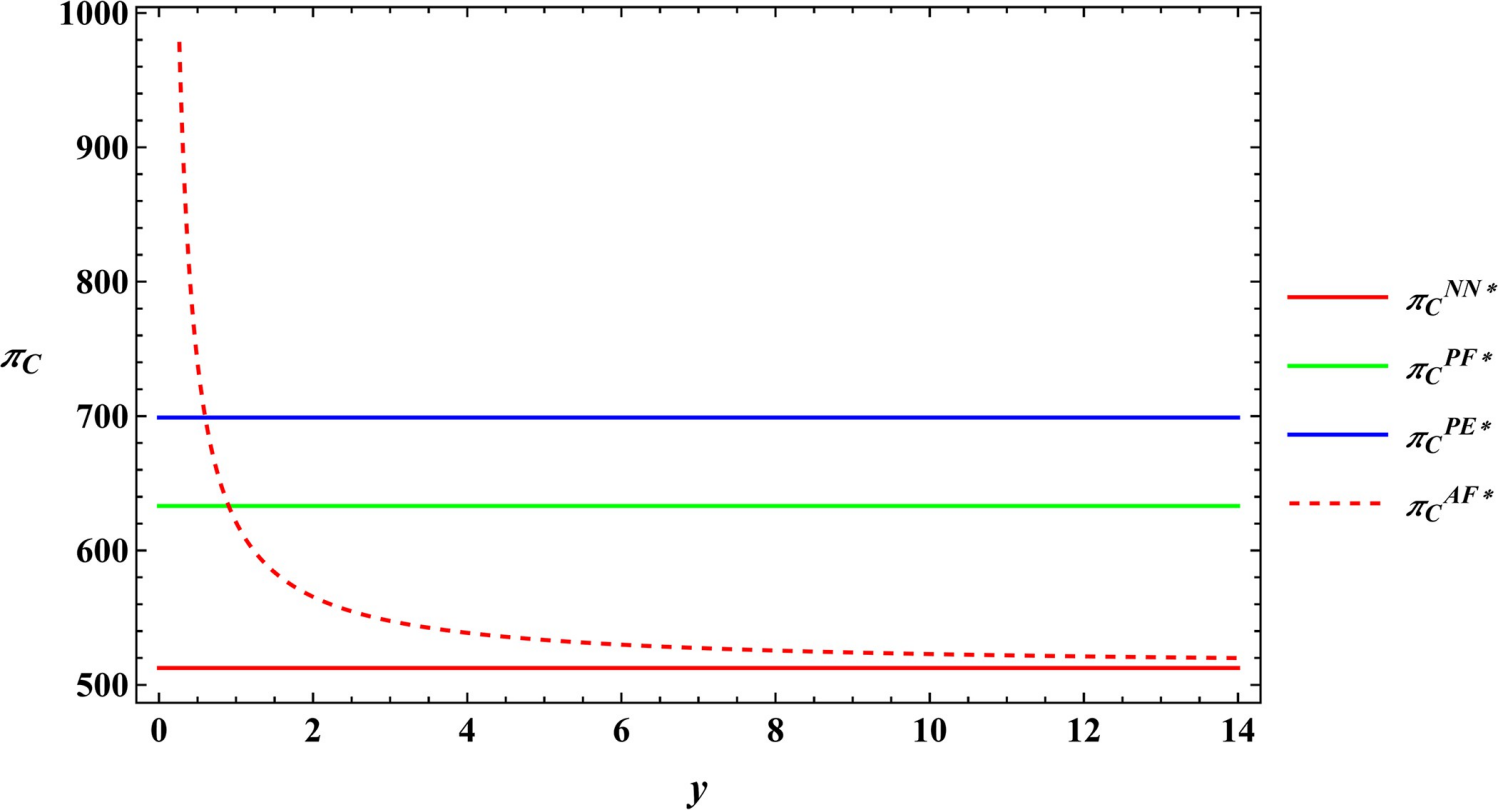
Comparative analysis of optimal profits for the e-commerce platform.

In the subsequent section, numerical simulations were conducted for a comparative analysis, with parameter settings consistent with those outlined in Proposition 2. The results are shown in Figs 19–22. The conclusions aligned with the aforementioned analysis, confirming the validity of the earlier model analysis. However, in the process, some interesting new insights have also been discovered.

First, the farm size is negatively related to agricultural productions per unit area, independent of the subsidy methods. The reason for this phenomenon is also easy to understand. When agricultural productions per unit area is higher, farmers can obtain sufficient agricultural products with less land, leading to a decrease in farm size as agricultural productions per unit area increase. Next, under both unsubsidized and price-subsidized models, the price of agricultural products, farmer’s profits, and e-commerce platform profits are independent of agricultural productions per unit area. In the context of both price subsidy and unsubsidized mode, the government subsidies method does not influence the purchase price of agricultural products. In the contract agriculture system, the quantity of farm products is predetermined before the purchase, resulting in the profits for the farmer and the e-commerce platform remaining unchanged. The final conclusion contradicts common knowledge. Under the area subsidy model, the purchase price of agricultural products will increase as the agricultural productions per unit area increases. At the same time, the profits of the farmer and the e-commerce platform will instead decrease. Under the area subsidy model, the government offers fixed-amount subsidies based on the cultivated area. Consequently, an increase in agricultural productions per unit area does not result in an increase in government subsidies. However, the heightened agricultural productions per unit area leads to increased inputs for farmers in terms of agricultural products. In response, farmers accept a relatively higher purchase price of agricultural products. The e-commerce platform has to receive a higher purchase price of agricultural products. At this point, farmers cannot generate a scale effect to mitigate the rising production costs, and if the government does not increase the subsidies amount, the farmer and the e-commerce platform experience a reduction in profits.

## 7. Conclusions

In this paper, the farmer participating in contract farming sell their agricultural products through the e-commerce platform. Consumers provide assistance to farmer through the e-commerce platform. This phenomenon is modeled and solved to examine the effects of consumer preferences for supporting farmers, consumer premiums, and different government subsidy models on the contract farming supply chain.

The main research conclusions of this paper are as follows: (1) Regardless of the type of government subsidy model, increasing consumer preference for supporting farmers and consumer payment premiums can help farmers expand their farm size, raise the purchase price of agricultural products, increase agricultural output, enhance the profits of both farmers and e-commerce platforms, and improve social welfare. (2) Farm size and the profits of both farmers and e-commerce platforms are positively correlated with government subsidies, regardless of the type of government subsidy model. The relationship between the purchase price of agricultural products and government subsidies depends on the subsidy target. When the government subsidy is directed at farmers, the purchase price of agricultural products decreases as government subsidies increase; when the subsidy is directed at e-commerce platforms, the purchase price of agricultural products increases with higher government subsidies. Additionally, under the price subsidy model, the correlation between social welfare and government subsidies is influenced by the government subsidy ratio and price elasticity; under the area subsidy model, the correlation between social welfare and government subsidies is affected by agricultural production per unit area. (3) The variations in farm size, the purchase price of agricultural products, and the profits of farmers and e-commerce platforms under different subsidy models are influenced by agricultural production per unit area. Regarding farm size, farmer profits, and e-commerce platform profits, as agricultural production per unit area increases, the optimal government subsidy model shifts from area subsidies to price subsidies targeted at e-commerce platforms. Concerning the purchase price of agricultural products, as agricultural production per unit area increases, the advantage of area subsidies diminishes.

Based on the above research conclusions, the following managerial implications can be drawn: (1) The government can use new media and other methods to promote agricultural products that support farmers, enhance consumer awareness and recognition of these products, and stimulate consumer interest in supporting agricultural products. (2) Different subsidy policies have their advantages and disadvantages for different stakeholders (farmer and e-commerce platform) under varying conditions. Therefore, in addition to focusing on social welfare objectives, the government can also consider trade-offs related to farm size, the purchase price of agricultural products, and the profits of the farmer and the e-commerce platform.

This paper differs from the previous studies by Peng et al. [5], Hong et al. [20], Xie et al. [40], and Sun et al. [46], and its difference are summarized in the following two main aspects. Firstly, in the current research on contract farming, few scholars have considered the impact of consumer preferences for supporting farmers and consumer premiums on the contract farming supply chain under the e-commerce assistance to farmers model. This paper, considering these two parameters, discusses the decision-making and profits of the farmer and the e-commerce platform. Thus, it further advances research in the field of consumer support for farmers. Secondly, most scholars have not differentiated government subsidies into specific types. This paper the changes in the contract farming supply chain under different government subsidy models were studied, and the impact of government subsidies on social welfare was considered. It provides reference significance for the government to adopt a more rational subsidy approach.

This study has certain limitations. For instance, in the e-commerce assistance to farmers model, the issue of supply chain coordination has not yet been explored. E-commerce platforms can collaborate with farmers to share costs, and further research could focus on the coordination of the contract farming supply chain under the e-commerce assistance to farmers model. It is hoped that this research will inspire further exploration of government subsidies within the supply chain of contract farming in the context of e-commerce assistance to farmers.
